# Self‐Management Interventions for Lower Urinary Tract Symptoms After Radical Prostatectomy: A Scoping Review of Intervention Components, Outcomes and Research Gaps

**DOI:** 10.1002/nop2.70991

**Published:** 2026-11-03

**Authors:** Koji Amano, Kumi Suzuki

**Affiliations:** ^1^ School of Nursing, Mukogawa Women's University Nishinomiya‐City Hyogo Japan; ^2^ Faculty of Nursing, Osaka Medical and Pharmaceutical University Takatsuki‐City Osaka Japan

**Keywords:** lower urinary tract symptoms, postoperative care, prostatectomy, self‐management interventions

## Abstract

**Aim:**

To map the existing literature on self‐management interventions for lower urinary tract symptoms (LUTS) following radical prostatectomy (RP) and clarify their characteristics and research gaps.

**Design:**

Scoping review.

**Review Methods:**

The study followed the Arksey and O'Malley methodological framework. Eligibility criteria included studies of adult men after RP that evaluated interventions incorporating self‐management components for LUTS. Data were extracted, categorized and synthesized according to intervention content.

**Data Sources:**

PubMed, CINAHL, PsycINFO and Web of Science were searched from their database inception to July 2026 to identify studies describing support programmes for patients undergoing RP.

**Results:**

Of the 480 identified records, 17 met the inclusion criteria. Interventions comprised lifestyle modification, follow‐up care and LUTS‐symptom management, delivered as standalone or combined approaches. Co‐interventions included pelvic‐floor muscle training, biofeedback, physical training and psychological counselling. Patients' self‐efficacy, particularly their perceived ability to control LUTS, was reported as a factor associated with promoting self‐management behaviours. While some studies reported improvements in key LUTS outcomes following the interventions, others found no significant between‐group differences or reported worsening of certain symptoms.

**Conclusion:**

Although self‐management support for LUTS following RP is diverse, this review identified inconsistencies in intervention components and outcome measures, as well as a lack of comparable interventional studies. Strategies to enhance motivation, symptom monitoring, lifestyle modification and education on pelvic floor muscle training were commonly incorporated into multifaceted self‐management interventions following RP.

**Implications for the Profession and/or Patient Care:**

Standardizing interventions description and adopting an LUTS‐specific outcome framework will strengthen the evidence base and help guide nurse‐led postoperative follow‐up models.

**Impact:**

Multifaceted and continuous self‐management interventions may support patients' ability to proactively manage postoperative LUTS and promote greater autonomy in daily life.

**Reporting Method:**

PRISMA‐ScR.

**Patient or Public Contribution:**

No patient or public contribution.

## 1 Introduction

Prostate cancer is the most commonly diagnosed cancer among men in approximately two‐thirds of countries worldwide (118 of 185 countries), and its incidence continues to increase annually (Bray et al. 2024). Radical prostatectomy (RP) is a standard treatment for localized prostate cancer, with many patients achieving favourable long‐term survival (Costello 2020). However, many patients undergoing RP experience lower urinary tract symptoms (LUTS), including urinary incontinence, increased urinary frequency and urinary urgency (Haga et al. 2015), which can persist long after treatment. Improvement beyond 12 months postoperatively is often difficult to achieve (Hikita et al. 2022; Kohada et al. 2024). Therefore, effective management of LUTS remains a major challenge in postoperative care to preserve quality of life (QOL).

Although LUTS due to RP do not directly affect survival, persistent symptoms have been associated with depression (Cheng et al. 2020), limitations in activities of daily living (Jones et al. 2023; Sun and Gu 2024), and increased financial burden (Lawless et al. 2025). Because improvement often requires long‐term treatment adherence (Chen et al. 2017), patients face a sustained burden. Patients may also be reluctant to seek help because of geographic barriers, financial difficulties, limited access to health care services and social norms discouraging emotional expression (Ettridge et al. 2018). Consequently, patients may experience insufficient support from health care professionals (Sun and Gu 2024), further complicating LUTS management.

The AUA/GURS/SUFU Guidelines (Breyer et al. 2024), the EAU Guidelines on Post‐Prostate Treatment Urinary Incontinence (European Association of Urology 2025), and a comprehensive review of guidelines (Bhatt et al. 2024) recommend conservative approaches, including pelvic floor muscle training (PFMT), patient education and counselling, as key components of managing post‐treatment urinary symptoms. Although these recommended management approaches are increasingly well established, little is known about how patients incorporate them into daily life and sustain them as part of self‐management. Furthermore, adherence to LUTS management behaviours has been identified as a key determinant of treatment outcomes following RP (Hodges et al. 2020).

Therefore, health care professionals have a responsibility to support patients in understanding their LUTS and maintaining the needed behaviours for effective self‐management. Following RP, patients should transition from passive self‐management during the early postoperative phase to active self‐management (Lawless et al. 2025). Few studies to date have investigated self‐management interventions for LUTS following RP, with the symptoms examined being predominantly focused on urinary incontinence. Moreover, intervention structures, intervention providers and evaluation criteria vary considerably. Research on patient‐led self‐management approaches for the complex and multifactorial nature of LUTS following RP remains limited, and the overall structure, content, and outcomes of these interventions remain unclear.

A scoping review is well‐suited to comprehensively map the extent, range and nature of research on a given topic, and to identify areas of convergence and divergence across studies, evidence gaps and priorities for future research (Arksey and O'Malley 2005; Peters et al. 2020). Using this approach to systematically describe and synthesize intervention components, including providers, tools, content, timing of assessment and associated outcomes, can clarify intervention characteristics, identify research gaps and provide insights for strengthening the evidence base. Such an overview can also inform the development and application of practical frameworks to guide future intervention design.

Therefore, this scoping review aimed to map and analyse the existing literature on self‐management interventions for LUTS in patients following RP and clarify the content of these interventions and the associated research gaps.

The findings of this study are expected to contribute to the systematization of self‐management support for LUTS in patients following RP and to advance intervention research and clinical nursing practice.

In this study, a self‐management intervention was defined as ‘a nursing intervention designed to enhance patients' ability to proactively manage LUTS following RP by supporting lifestyle modification, symptom management—including coping with LUTS and LUTS‐related challenges—and sustained self‐care behaviors’. Interventions consisting solely of conventional conservative management, such as PFMT or biofeedback delivered in isolation, were not considered self‐management interventions unless they included additional components designed to promote patients' active participation in self‐management.

## 2 Methods

### 2.1 Information Sources and Search Strategy

This scoping review followed the methodological framework proposed by Arksey and O'Malley (2005) and was reported in accordance with the Preferred Reporting Items for Systematic Reviews and Meta‐Analyses Extension for Scoping Reviews (PRISMA‐ScR) (Tricco et al. 2018). The completed PRISMA‐ScR checklist is provided in Table S1.

Literature searches were performed in PubMed, CINAHL, PsycINFO and Web of Science. The search strategy was developed to identify studies examining self‐management interventions among patients with prostate cancer who underwent RP. The search strategy was guided by the Population, Concept, Context (PCC). The population comprised adult patients who underwent RP for prostate cancer. Studies focusing exclusively on preoperative patients or interventions delivered only before RP were excluded. The concept focused on self‐management interventions for LUTS. The context encompassed postoperative management of LUTS following RP across various healthcare settings, including hospitals, outpatient clinics, community‐based care and home care. Search terms included combinations of terms such as RP, prostate cancer, surgery, self‐management, symptom management, self‐care and LUTS.

This review included empirical studies that evaluated or described interventions incorporating self‐management components for LUTS following RP. Eligible study designs included randomized controlled trials (RCTs), quasi‐experimental designs (QEDs) and observational studies evaluating these interventions in clinical practice. The search was limited to articles published in English. Conference abstracts and commentaries were excluded to ensure the synthesis of original research findings. The literature search was conducted on July 6, 2026. Full search strategies for each database are provided in Table S2.

### 2.2 Screening and Study Selection Criteria

After duplicates were removed using Rayyan (Ouzzani et al. 2016), studies describing support programmes for managing LUTS following RP were screened and selected according to the eligibility criteria. Rayyan enabled independent screening by multiple reviewers, identification of discrepancies and systematic documentation of reasons for exclusion, thereby enhancing the transparency and reproducibility of the study selection process.

Regarding study design, we excluded methodological reports, narrative reviews, study protocols, conference abstracts, commentaries and observational studies that did not evaluate the effects of self‐management or nursing interventions. We also excluded studies involving populations other than patients with prostate cancer following RP, studies primarily evaluating the efficacy of additional treatments following RP, and studies focusing on post‐treatment outcomes unrelated to LUTS management.

Title/abstract and full‐text screening were conducted by the first author (A.K.). Subsequently, to ensure consistency and validity in study selection, the second author (S.K.) reviewed the screening decisions, eligibility assessments, reasons for exclusion, and their consistency with the predefined eligibility criteria. Cases requiring clarification were discussed and re‐examined by the authors until consensus was reached. The study selection process was summarized in a PRISMA flow diagram, which also details the reasons for exclusion at the full‐text review stage. Consistent with scoping review methodology, no formal assessment of study quality or risk of bias was performed.

### 2.3 Data Analysis and Synthesis

A data charting form was developed to systematically organize the included studies and record key study characteristics. Extracted data included: (1) publication details, including author(s), year of publication and country; (2) study design, intervention components, intervention tools, outcome measures and assessment timing; (3) participant characteristics, including sample size, group allocation, age and nerve‐sparing status or other perioperative factors; and (4) intervention outcomes. Data charting was conducted in duplicate.

Extracted data were categorized according to the type of intervention and LUTS addressed, with the results summarized in tables. Where applicable, outcome measures were grouped into five domains to facilitate clinical interpretability (lower urinary tract function, QOL, pelvic floor muscle function, psychological outcomes and self‐efficacy and clinical course). Self‐management support components were identified, categorized by component and organized in relation to the outcomes. As this review did not involve human participants, institutional ethics review board approval was not required.

## 3 Results

### 3.1 Search Results

A total of 480 records were identified through the database searches. After duplicate removal, 431 records remained for title and abstract screening, of which 393 were excluded. Full‐text assessment of the remaining 38 records resulted in the exclusion of 21 studies. The final analysis included 17 studies, with the selection process illustrated in the PRISMA flow diagram (Figure 1).

**FIGURE 1:**
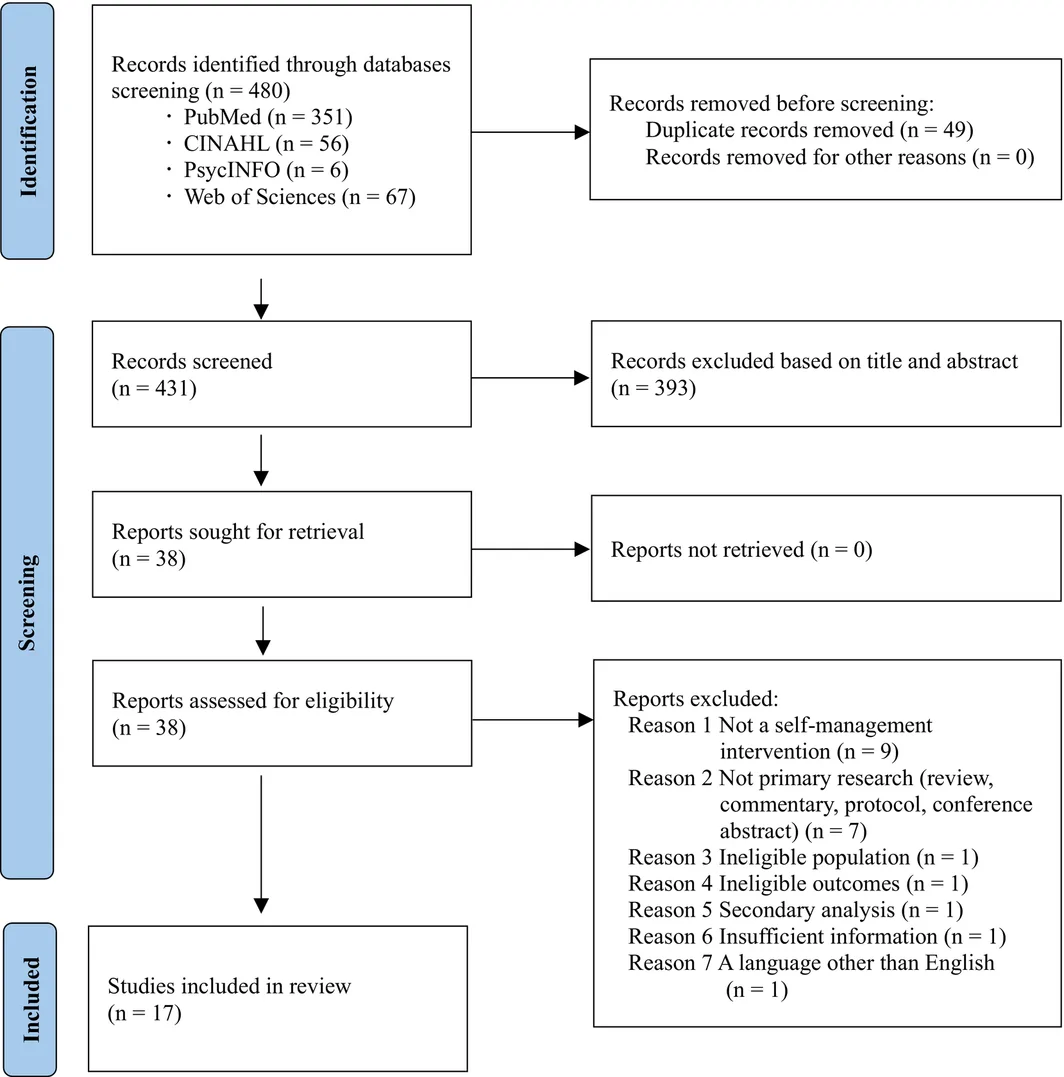
PRISMA flow diagram.

### 3.2 Study Characteristics

An overview of the 17 included studies is presented in Table 1. China accounted for the largest number of studies (*n* = 8), followed by the United States (*n* = 3). One study was conducted in Australia, Brazil, Japan, Sweden, Taiwan and Turkey.

**TABLE 1:** Study characteristics.

Author (year), country	Study design	Sample size	Participant characteristics	Care provider	Follow‐up period	Outcome domains
Fan et al. (2026), China	Pilot feasibility study	16	Mean age 67.6 ± 4.3 years (range 61–74 years)	Health care team (physicians and nurses)	Baseline, 3 months after intervention	LUTS function: Bladder diary parameters, 1‐h pad test, International Consultation on Incontinence Questionnaire‐Short Form (ICIQ‐UI SF)
Xu et al. (2025), China	Retrospective cohort study	165	After PSM: Observation group 63.4 ± 5.9 years; Control group 63.1 ± 5.3 years	Trained nurse	Baseline, 2 weeks, 1 month, 3 months post‐surgery	LUTS function: International Consultation on Incontinence Questionnaire‐Urinary Incontinence Short Form (ICIQ‐UI SF), urinary incontinence incidence QOL: Functional Assessment of Cancer Therapy‐Prostate (FACT‐P) Psychological outcomes and self‐efficacy: Self‐Rating Anxiety Scale (SAS), Self‐Rating Depression Scale (SDS) Clinical course: Postoperative complications
Chien et al. (2025), Taiwan	Experimental, two‐group parallel design using block randomization (RCT)	76	Intervention group: 67.2 ± 6.5 years; Control group: 68.5 ± 6.0 years	Trained nurse	Baseline, 12 weeks, 16 weeks	LUTS function: Single‐item questionnaire for urinary incontinence severity QOL: Expanded Prostate Cancer Index Composite (EPIC), Social participation scale for elderly adults Psychological outcomes and self‐efficacy: Traditional Chinese version of the cancer survivors' self‐efficacy scale, Chinese version of the 10‐item Connor‐Davidson Resilience Scale (CD‐RISC), Chinese version of the demoralization scale, level of confidence in self‐management
Gerlegiz et al. (2025), Turkey	Three‐group parallel randomized controlled trial (RCT)	66	Group I (pelvic floor muscle training (PFMT) + Knack + lifestyle): 62.3 ± 6.9 years; Group II (PFMT + Knack): 66.0 ± 8.4 years; Group III (control, PFMT only): 64.6 ± 5.1 years	n/a	Baseline, after intervention (8 weeks)	LUTS function: International Consultation on Incontinence Questionnaire‐Short Form (ICIQ‐UI SF), 1‐h pad test, Patient Global Impression of Severity Scale (PGI‐S) QOL: King's Health Questionnaire (KHQ), Patient Global Impression of Improvement Scale (PGI‐I) Pelvic floor muscle function: Adherence to pelvic floor muscle exercise Clinical course: Turkish‐International Physical Activity Questionnaire‐Short Form (IPAQ‐SF), Adverse events
Heneka et al. (2024), Australia	Single‐group quasi‐experimental (pre–post) design combined with exploratory qualitative investigation	17	Single‐group study: 64.0 ± 5.0 years	n/a	T1—prior to intervention commencing (baseline); T2—at completion of the 12‐week intervention; and T3—3 months post‐intervention completion	QOL: Expanded Prostate Cancer Index Composite (EPIC), The European Quality of Life‐5 Dimension 5‐Level (EQ‐5D‐5L) Psychological outcomes and self‐efficacy: eHealth Literacy Scale (eHEALS), Distress Thermometer and Problem List validated for prostate cancer, Insomnia Severity Index, Brief Fatigue Inventory (BFI) Clinical course: Godin‐Shephard Leisure‐Time Physical Activity Questionnaire (GSLTPAQ)
Gu et al. (2024), China	Retrospective cohort study	228	Intervention group: 67.8 ± 7.2 years; Control group: 66.8 ± 7.5 years	Incontinence specialist nurse	Day of catheter removal, 1 month, 3 months, 6 months and 12 months or more postoperatively	LUTS function: 24‐h urine pad test, International Consultation on Incontinence Questionnaire‐Short Form (ICIQ‐UI SF) Pelvic floor muscle function: Glazer evaluation method for assessing pelvic floor muscle function
Yang et al. (2024), China	Retrospective cohort study	189	Intervention group: 56.9 ± 5.8 years; Control group: 56.8 ± 5.7 years	Head nurse and nurses	Day 1 postoperatively, at discharge (more than 2 weeks later)	QOL: SF‐36 Psychological outcomes and self‐efficacy: Disease‐related knowledge, Evaluation Scale for Self‐Care Ability (ESCA), Hamilton Anxiety Scale (HAMA), Hamilton Depression Scale (HAMD) Clinical course: Urethral catheterization duration and incontinence duration
Xin and Guo (2024), China	Prospective RCT	66	Comprehensive intervention group: 63.2 ± 2.4 years (range 56–68); Standard care group: 62.3 ± 2.4 years (range 57–68)	Nurse	At admission, after nursing intervention	LUTS function: 1‐h urine pad method QOL: European Organization for Research and Treatment of Cancer Core Quality of Life Scale Psychological outcomes and self‐efficacy: Self‐Rating Anxiety Scale (SAS), Self‐Efficacy Scale Clinical course: Postoperative hospitalization period
Wang et al. (2024), China	RCT	94	Intervention group: age not reported; Standard care group: age not reported	Nurse, physician	Before care, after care	LUTS function: International Prostate Symptom Score (IPSS), International Consultation on Incontinence Questionnaire‐Short Form (ICIQ‐UI SF) QOL: SF‐12 Health Survey Psychological outcomes and self‐efficacy: Prostate Cancer Disease Awareness Questionnaire, Self‐Rating Anxiety Scale (SAS), Chinese Version of the Self‐Reported ‘Strategies for Promoting Health’ (SUPPH) Clinical course: Health Care Adherence Behaviour
Wennerberg et al. (2023), Sweden	RCT	165	Intervention group: 64.0 ± 6.2 years; Control group: 64.0 ± 6.3 years	Cancer nurse specialist	1, 3, 6, 12 months post‐surgery	QOL: Expanded Prostate Cancer Index Composite (EPIC) Pelvic floor muscle function: Postoperative pelvic floor muscle exercise frequency Clinical course: Saltin‐Grimby Physical Activity Level Scale, ePATH usage history
Izidoro et al. (2022), Brazil	Single‐blind clinical trial using 1:1 randomization (RCT)	34	Intervention group: age not reported; Control group: age not reported	n/a	15–20 days after removal of indwelling urinary catheter (Baseline ‐ T0); 30 days after baseline (T1); 60 days after baseline (T2); and 90 days after baseline (T3)	LUTS function: Pad test, Urinary Incontinence Scale for Radical Prostatectomy (UISRP) QOL: King's Health Questionnaire (KHQ)
Song (2021), China	Retrospective cohort study	100	Experimental group: 76.4 ± 2.7 years; Control group: 75.2 ± 2.6 years	Nurse	Not clearly reported	LUTS function: Urinary incontinence incidence, urinary control effectiveness rate QOL: WHO Quality of Life Scale Psychological outcomes and self‐efficacy: Exercise of Self‐Care Agency Scale (ESCA) Clinical course: Complication incidence
Sato (2020), Japan	Single‐group intervention study (QED)	30	Single‐group study: 62.7 ± 4.1 years	Nurse, physician	1 month, 3 months post‐surgery	LUTS function: Pad Test, Measurement of Residual Urine in the Bladder QOL: Expanded Prostate Cancer Index Composite (EPIC)
Yuan et al. (2019), China	RCT	35	Intervention group: 71.2 ± 5.5 years; Control group: 73.1 ± 5.1 years	Team consisting of physicians and nurses	Baseline, 3 months	QOL: Incontinence‐Specific Quality of Life Scale (I‐QOL) Psychological outcomes and self‐efficacy: Self‐Rating Anxiety Scale (SAS), Self‐Rating Depression Scale (SDS)
Zhang et al. (2015), United States	RCT	279	Support group: 66.8 ± 7.2 years; Telephone group: 64.3 ± 7.3 years; Usual care group: 64.9 ± 8.2 years	Nurse specialist, health psychologists	Baseline, 3 months, 6 months	LUTS function: 1‐h pad test, International Prostate Symptom Score (IPSS), UCLA Prostate Cancer Index urinary function and bother subscales QOL: Disease‐specific quality of life (UCLA‐PCI), VAS rating of incontinence bother
Goode et al. (2011), United States	Multicenter RCT	208	Behaviour plus group (behavioural therapy + biofeedback + pelvic floor muscle electrical stimulation): 66.8 ± 7.0 years; Behaviour group (behavioural therapy alone): 66.3 ± 7.3 years; Control group: 66.9 ± 7.7 years	Nurse or physician	8 weeks, 6 months, 12 months post‐surgery	LUTS function: Reduction rate in urinary incontinence episodes based on bladder diary, AUA‐7 Symptom Score, International Prostate Symptom Score (IPSS) QOL: Incontinence Impact Questionnaire (IIQ), Expanded Prostate Cancer Index Composite (EPIC), SF‐36, Global Perceived Improvement Scale, Patient Satisfaction Questionnaire
Robinson et al. (2008), United States	RCT	126	Intervention group: 60.2 ± 6.4 years; Control group: 59.2 ± 6.7 years	Advanced practice nurse	3, 6, 12 months post‐surgery	LUTS function: Male Urogenital Distress Inventory (MUDI) QOL: Male Urinary Symptom Impact Questionnaire (MUSIQ)

Of the 17 studies, 10 were RCTs, four were retrospective cohort studies, two were QEDs, and one was a pilot feasibility study. Both QEDs used a single‐group design; one was a before‐and‐after study combined with qualitative exploration, and the other was a single‐group intervention study.

Within the lower urinary tract function domain, outcomes were commonly assessed using the pad test (Fan et al. 2026; Gerlegiz et al. 2025; Gu et al. 2024; Izidoro et al. 2022; Sato 2020; Xin and Guo 2024), the International Consultation on Incontinence Questionnaire‐Short Form (ICIQ‐SF) (Fan et al. 2026; Gu et al. 2024; Gerlegiz et al. 2025; Wang et al. 2024; Xu et al. 2025) and the International Prostate Symptom Score (IPSS) (Goode et al. 2011; Wang et al. 2024; Zhang et al. 2015). QOL outcomes were evaluated using the Short Form Health Survey (SF‐36/SF‐12) (Goode et al. 2011; Wang et al. 2024; Yang et al. 2024), the Expanded Prostate Cancer Index Composite (EPIC) (Chien et al. 2025; Goode et al. 2011; Heneka et al. 2024; Sato 2020; Wennerberg et al. 2023), the King's Health Questionnaire (KHQ) (Gerlegiz et al. 2025; Izidoro et al. 2022), the University of California, Los Angeles Prostate Cancer Index (UCLA‐PCI) (Zhang et al. 2015) and the Incontinence‐Specific QOL Scale (I‐QOL) (Yuan et al. 2019). Pelvic floor muscle function was assessed by PFMT practice frequency (Wennerberg et al. 2023), adherence to PFMT (Gerlegiz et al. 2025) and the Glazer Assessment Scale for evaluating pelvic floor muscles (Gu et al. 2024). Psychological outcomes and self‐efficacy were assessed using the Self‐Rating Anxiety Scale (SAS), Self‐Rating Depression Scale (SDS), Hamilton Anxiety Scale (HAMA), Hamilton Depression Scale (HAMD) (Wang et al. 2024; Xin and Guo 2024; Xu et al. 2025; Yang et al. 2024; Yuan et al. 2019), Self‐Efficacy Scale (Chien et al. 2025; Xin and Guo 2024) and the level of confidence in self‐management (Chien et al. 2025), among others. Clinical course outcomes included adverse events (Gerlegiz et al. 2025), complications (Xu et al. 2025; Song 2021), duration of catheterization and duration of urinary incontinence (Yang et al. 2024).

### 3.3 Participant Characteristics

Sample size across the included studies ranged from 16 to 279 participants, with a mean sample size of 111.4. Participants' mean age ranged from 56.8 to 76.4 years. Among the four studies reporting nerve‐sparing status, the neurovascular bundle (NVB) preservation rate was 64.4% (293 out of 455 participants).

### 3.4 Self‐Management Intervention for LUTS Following RP

Table 2 summarizes the intervention content. The included interventions comprised lifestyle modification (*n* = 15), follow‐up care (*n* = 12) and LUTS symptom management (*n* = 13). These forms of support were delivered as standalone interventions or in combination. Additional interventions implemented included PFMT (*n* = 15), biofeedback (*n* = 4), physical training (*n* = 2) and psychological counselling (*n* = 2). Lifestyle modification components comprised smoking cessation (Gerlegiz et al. 2025; Gu et al. 2024; Izidoro et al. 2022), alcohol reduction (Gerlegiz et al. 2025; Gu et al. 2024; Sato 2020), fluid intake management (Fan et al. 2026; Gerlegiz et al. 2025; Gu et al. 2024; Xin and Guo 2024; Izidoro et al. 2022; Sato 2020; Zhang et al. 2015; Goode et al. 2011; Robinson et al. 2008), restriction of caffeinated beverages (Fan et al. 2026; Gerlegiz et al. 2025; Gu et al. 2024; Izidoro et al. 2022; Zhang et al. 2015; Goode et al. 2011), avoidance of foods containing bladder irritants (Gu et al. 2024; Izidoro et al. 2022), nutritional management (Fan et al. 2026; Gu et al. 2024; Xin and Guo 2024; Gerlegiz et al. 2025; Yang et al. 2024; Song 2021; Zhang et al. 2015; Robinson et al. 2008), weight management (Gerlegiz et al. 2025; Gu et al. 2024), bladder training (Fan et al. 2026; Gu et al. 2024; Yang et al. 2024; Yuan et al. 2019; Zhang et al. 2015; Goode et al. 2011; Robinson et al. 2008), adjustment of voiding habits (Fan et al. 2026; Xu et al. 2025; Gu et al. 2024; Xin and Guo 2024; Goode et al. 2011) and promotion of physical activity (Zhang et al. 2015; Chien et al. 2025; Wang et al. 2024; Gerlegiz et al. 2025; Yang et al. 2024). Follow‐up care included individualized patient follow‐up, with or without family involvement (Fan et al. 2026; Chien et al. 2025; Song 2021; Sato 2020; Gu et al. 2024; Zhang et al. 2015; Yuan et al. 2019; Wang et al. 2024; Izidoro et al. 2022; Robinson et al. 2008). Delivery modalities included face‐to‐face support (Gerlegiz et al. 2025; Goode et al. 2011; Gu et al. 2024; Heneka et al. 2024; Izidoro et al. 2022; Robinson et al. 2008; Wang et al. 2024; Xin and Guo 2024; Xu et al. 2025; Yang et al. 2024; Yuan et al. 2019; Zhang et al. 2015), remote support delivered via telephone or online communication (Chien et al. 2025; Izidoro et al. 2022; Robinson et al. 2008; Sato 2020; Wang et al. 2024; Yuan et al. 2019; Zhang et al. 2015) and digital interventions delivered via web‐based, mobile or app‐based platforms (Chien et al. 2025; Fan et al. 2026; Sato 2020; Song 2021; Wang et al. 2024; Wennerberg et al. 2023).

**TABLE 2:** Summary of self‐management interventions for LUTS after RP.

Author	Type	Components of intervention	Mode and intensity	Main findings
Gu et al. (2024)	Lifestyle modification Follow‐up care Symptom management ○ Pelvic floor muscle training ○ Biofeedback	Postoperative care and lifestyle guidance Assessment and education on urinary management Symptom monitoring and specialist referral Pelvic floor muscle training with biofeedback	Stage‐based face‐to‐face follow‐up over ≥ 12 months	The intervention group showed significantly lower 24‐h pad test results than the control group at 2 and 6 months postoperatively. In addition, the Incontinence Questionnaire–Short Form (ICIQ‐SF) scores in the intervention group (IG) were significantly lower than those in the control group (CG) at 1 month postoperatively
Zhang et al. (2015)	Lifestyle modification Follow‐up care Symptom management ○ Pelvic floor muscle training ○ Biofeedback	Lifestyle and bladder management Biofeedback‐assisted pelvic floor muscle training Goal‐setting and adherence support Symptom self‐management training Peer or telephone‐based support	Face‐to‐face peer support or telephone‐based intervention	At 3 months, both intervention groups had a lower frequency of daily urinary leakage than the usual‐care group. At 6 months, symptom severity and incontinence‐related problems were lower in both intervention groups; participants in the support group also had less urine leakage, whereas the telephone group reported better urinary function and less bother
Fan et al. (2026)	Lifestyle modification Follow‐up care Symptom management ○ Pelvic floor muscle training	Lifestyle and continence care education Pelvic floor muscle and bladder training Symptom monitoring and self‐management support Personalized feedback with clinician support Reinforcement learning (RL)‐based digital self‐management intervention	App‐based digital therapeutic intervention (mean use: 5.2 times/day; 3 months)	After the 3‐month intervention, significant improvements were observed in urinary frequency, incontinence episodes, urine leakage, ICIQ‐UI SF scores, urinary urgency, incomplete bladder emptying and dysuria. No significant improvement was observed in postvoid dribbling
Chien et al. (2025)	Lifestyle modification Follow‐up care Symptom management ○ Pelvic floor muscle training	Educational content (self‐management, social participation) Pelvic floor muscle training monitoring Push notifications to support adherence (e.g., pelvic floor muscle training) Self‐efficacy assessment and support Mobile self‐management app (education and monitoring) Weekly nurse telephone support	App‐based intervention with weekly nurse support (≥ 5 min/session)	The IG showed significant improvements at both 12 and 16 weeks compared with baseline in urinary incontinence (UI), irritative/obstructive bladder symptoms, participation in activities, motivation and satisfaction regarding social participation and self‐efficacy. Compared with the CG, the IG showed significant improvement in irritative and obstructive bladder symptoms at Week 12 and 16, there were significant improvements in participation in interpersonal activities, motivation for social participation and satisfaction with social participation
Izidoro et al. (2022)	Lifestyle modification Follow‐up care Symptom management ○ Pelvic floor muscle training	Cognitive–behavioural therapy (CBT) nursing intervention Booklet‐based and lifestyle education Progressive pelvic floor muscle training programme Behavioural and motivational support Symptom monitoring Telephone and text‐based follow‐up	Face‐to‐face, telephone and text‐based follow‐up (12‐week) PFMT programme	Pad test results showed a reduction in the severity of post‐radical prostatectomy UI in the IG, with a significant difference compared with the CG at the first month of follow‐up (T1). When assessed using the Urinary Incontinence Scale of Radical Prostatectomy, UI severity was also significantly reduced in the IG at all follow‐up time points. Regarding the group × time interaction, significant differences between the IG and CG were observed at the end of follow‐up for urinary frequency, urinary urgency, nocturia and UI
Song (2021)	Lifestyle modification Follow‐up care Symptom management ○ Pelvic floor muscle training	Health education and self‐management support Pelvic floor muscle training Symptom monitoring Individualized follow‐up and psychosocial support Network‐based continuous nursing intervention	Network‐based continuous nursing with online follow‐up (≥ 3 days/week)	At post‐intervention assessment, the experimental group had a lower incidence of urinary incontinence and a higher urinary control effectiveness rate than the control group. Self‐care ability and quality of life were higher, whereas complication rates were lower
Sato (2020)	Lifestyle modification Follow‐up care Symptom management ○ Pelvic floor muscle training	Education on pelvic floor muscle training, fluid intake and medication Web‐based self‐management with symptom monitoring Communication via email or chat Automated data visualization for feedback Cloud‐based monitoring and data sharing	Web‐based self‐management with continuous monitoring	Scores for UI and voiding dysfunction were significantly higher at 3 months than at 1 month postoperatively, indicating symptom improvement
Yuan et al. (2019)	Lifestyle modification Follow‐up care Symptom management ○ Physical training	Routine care and psychoeducation by a multidisciplinary team Multimodal patient and family education Monitoring and feedback on self‐care Rehabilitation and functional exercise support Addressing negative emotions and complaints Weekly telephone follow‐up	Face‐to‐face intervention with weekly telephone follow‐up	At 3 months after urethral catheter removal, the psychoeducation group had significantly lower Self‐Rating Anxiety Scale (SAS) and Self‐Rating Depression Scale (SDS) scores than the conventional nursing group. At the same time, the psychoeducation group showed significantly higher Incontinence‐Specific QoL Scale scores compared with the conventional nursing group
Robinson et al. (2008)	Lifestyle modification Follow‐up care ○ Pelvic floor muscle training ○ Biofeedback	Lifestyle education and feedback Behavioural strategies for urinary control Biofeedback‐assisted pelvic floor muscle training Ongoing structured instruction on pelvic floor muscle control and techniques Home‐based pelvic floor muscle training with adherence monitoring	Weekly home visits with telephone follow‐up and daily exercises (3 sets/day)	At all data collection points, strong positive correlations were observed among LUTS severity, LUTS‐related distress and HRQL. Although both the intervention and control groups showed significant reductions in LUTS severity and LUTS‐related distress within 12 months postoperatively, no significant between‐group differences were observed at 3, 6 or 12 months
Wang et al. (2024)	Lifestyle modification Follow‐up care ○ Pelvic floor muscle training	Postoperative WeChat‐based education (pelvic floor muscle training, lifestyle, complications) Behavioural and adherence support Individualized assessment and planning Support for enhancing self‐efficacy Psychosocial support	Face‐to‐face, telephone and WeChat‐based follow‐up (5–10 min/session)	After the intervention, the Information–Knowledge–Attitude–Practice (IKAP) theory–based group showed significantly greater reduction in IPSS scores compared with the standard care group. Similarly, ICIQ‐SF scores in the IKAP group were significantly lower than those in the standard care group
Xu et al. (2025)	Lifestyle modification Symptom management ○ Pelvic floor muscle training ○ Counselling	Patient education and follow‐up Symptom management theory‐based nursing intervention Pelvic floor muscle training Symptom assessment and individualized management Psychological support and counselling	Face‐to‐face symptom management programme (2 weeks)	At the end of treatment and at 1 month post‐treatment, the observation group had lower urinary incontinence severity, pain, anxiety and depression scores than the control group. Urinary incontinence incidence and 3‐month complication rates were lower, whereas FACT‐P scores were higher in the observation group
Goode et al. (2011)	Lifestyle modification Symptom management ○ Pelvic floor muscle training ○ Biofeedback	Comprehensive lifestyle modification Pelvic floor muscle training‐based behavioural therapy Bladder training and urge control Self‐monitoring with bladder diaries Biofeedback and electrical stimulation	Four face‐to‐face visits over 8 weeks with daily home exercises	At 8 weeks, the mean reduction in weekly incontinence episodes was significantly greater in the behaviour plus group than in the control group. The behaviour plus group did not have improved 8‐week outcomes compared with the behaviour group. However, this intervention resulted in significantly greater mean reduction in weekly incontinence episodes than the control group. No significant difference was observed between the behaviour group and the behaviour plus group. At 8 weeks, urinary domain and UI scores showed significantly greater improvements in both the behaviour group and behaviour plus group compared with the control group
Xin and Guo (2024)	Lifestyle modification Symptom management ○ Pelvic floor muscle training	Perioperative education and support Comprehensive nursing care Symptom and complication management Pelvic floor muscle training‐based rehabilitation and recovery support Motivational and confidence‐building support	Face‐to‐face perioperative nursing care during hospitalization	At 6 months after the intervention, UI scores in the comprehensive group were significantly better than those in the standard group. The SAS and SDS scores were also significantly lower in the comprehensive group than in the standard group. In addition, self‐care ability, health knowledge level and self‐care responsibility were all significantly higher in the comprehensive group than in the standard group
Wennerberg et al. (2023)	Follow‐up care Symptom management ○ Pelvic floor muscle training ○ Physical training	App/web‐based self‐management platform (ePATH) Personalized self‐care modules tailored to patient needs Pelvic floor muscle training and physical activity guidance Self‐monitoring, goal setting and progress tracking Educational content and reminders Asynchronous nurse communication	Self‐directed digital intervention with daily use	Urinary continence scores were significantly higher at 12 months than at 1, 3 and 6 months postoperatively, indicating improvement. No significant difference in UI scores was observed between the IG and CG. Compared with the preoperative levels, UI scores worsened at 1 month postoperatively and had not returned to the preoperative levels by 12 months, indicating persistent deterioration. The ePATH intervention showed no significant effect on physical activity, and no significant difference was observed between the IG and CG in the performance of pelvic floor muscle training
Heneka et al. (2024)	Follow‐up care Symptom management	CBT‐based self‐management intervention Psychoeducation and symptom management Stress management and problem‐solving Goal setting support	Weekly CBT‐based sessions	From baseline (T1) to 3 months (T3), the EPIC HRQoL scores for irritative/obstructive urinary symptoms showed significant improvement, and the perceived urinary burden over the past 4 weeks also decreased. However, no significant differences were observed for UI, nocturia or urinary frequency
Yang et al. (2024)	Lifestyle modification ○ Pelvic floor muscle training ○ Counselling	Lifestyle and behavioural guidance Pelvic floor muscle training‐based rehabilitation Adherence support and reinforcement of self‐care behaviours CBT‐based nursing intervention Psychological and educational support Psychological counselling	Daily face‐to‐face intervention (40 min) with home exercises (2–3 sets/day)	The duration of urethral catheterization and the duration of UI did not differ between the observation and control groups. At discharge, disease knowledge and self‐care scores were significantly higher in the observation group than in the control group, despite being comparable on postoperative Day 1. Similarly, the Hamilton Anxiety Scale and Hamilton Depression Scale scores did not differ between the groups on postoperative Day 1, but at discharge, the scores in the observation group were significantly lower than those in the control group
Gerlegiz et al. (2025)	Lifestyle modification ○ Pelvic floor muscle training	Pelvic floor muscle training incorporating the Knack manoeuvre Integration of pelvic floor contraction into functional movements (e.g., coughing, lifting) Supervised instruction with feedback to ensure correct technique Lifestyle modification and education	Supervised in‐clinic training with daily application during routine activities	At Week 8, ICIQ‐UI SF scores improved significantly in Group I (Pelvic floor muscle training + Knack + lifestyle group) compared with the other groups. In addition, urine leakage volume measured by the 1‐h pad test was significantly lower in Group I than in the other groups. Significant improvements were also observed in Group I in the subdomains of impact of incontinence, role limitations, physical limitations, limitations in personal relationships and emotional problems

Abbreviations: CBT, cognitive behavioural therapy; CG, control group; EPIC, expanded prostate cancer index composite; IG, intervention group; LUTS, lower urinary tract symptoms; SAS, self‐rating anxiety scale; SDS, self‐rating depression scale; UI, urinary incontinence.

### 3.5 Outcomes by Type of Self‐Management Interventions

#### 3.5.1 Interventions Combining Lifestyle Modification, Follow‐Up Care and Symptom Management

Eight studies reported interventions combining all three self‐management components: lifestyle modification, follow‐up care and LUTS‐focused symptom management (Chien et al. 2025; Fan et al. 2026; Gu et al. 2024; Izidoro et al. 2022; Sato 2020; Song 2021; Yuan et al. 2019; Zhang et al. 2015). As part of follow‐up care, nurses provided counselling to address patients' concerns regarding daily life and facilitated individualized adjustments (Gu et al. 2024; Izidoro et al. 2022; Sato 2020; Song 2021; Yuan et al. 2019; Zhang et al. 2015). Symptom management components included active symptom monitoring and the provision of feedback (Chien et al. 2025; Fan et al. 2026; Gu et al. 2024; Izidoro et al. 2022; Sato 2020; Song 2021; Yuan et al. 2019). Tools used to support follow‐up care and symptom management included self‐management apps (Chien et al. 2025; Fan et al. 2026), tablet‐based telehealth (Sato 2020) and network cloud platforms (Song 2021).

A single‐group study examining changes over time reported significant improvements in urinary incontinence and the subjective burden associated with urination at 3 months postoperatively compared with 1 month postoperatively (Sato 2020). Another single‐group study of a similar digital therapeutic intervention reported improvements in urinary incontinence episodes, urine leakage volume, urinary frequency, urinary urgency, incomplete bladder emptying and symptom burden (Fan et al. 2026). One study evaluating nurse‐led follow‐up care reported significantly greater improvement in urinary incontinence severity in the intervention group than in the control group at 1–2 months postoperatively; the intervention included educational components to enhance self‐efficacy, motivational strategies using social persuasion and vicarious experience, and symptom monitoring (Izidoro et al. 2022). Furthermore, compared with the control group, the intervention group demonstrated improvements in cancer‐related self‐management (Chien et al. 2025), irritative and obstructive urinary symptoms (Chien et al. 2025; Izidoro et al. 2022), and storage symptoms—including urinary incontinence, frequency and urgency (Gu et al. 2024; Izidoro et al. 2022; Song 2021), motivation and satisfaction related to social participation (Chien et al. 2025), QOL (Song 2021; Zhang et al. 2015) and anxiety and depression (Yuan et al. 2019).

However, one study reported no significant difference between intervention and control groups in terms of urinary incontinence severity or participation in leisure, sports and religious activities (Chien et al. 2025). These findings suggest variability across studies depending on the outcome assessed, underlining a lack of consistency in the reported results.

#### 3.5.2 Interventions Combining Lifestyle Modification and Follow‐Up Care

Two studies incorporating instructions in PFMT examined interventions combining lifestyle modification and follow‐up care (Robinson et al. 2008; Wang et al. 2024). One study described follow‐up care focused on helping patients develop appropriate medical understanding and confidence in self‐training by analysing factors that influence self‐care, providing guidance to family members and fostering patient confidence through supportive interactions (Wang et al. 2024). The intervention incorporated action planning, behaviour recording and integration of self‐management behaviours into daily life to promote behaviour change. Compared with standard care, the intervention group demonstrated significantly greater improvement in LUTS at 3 months, along with reductions in anxiety and depression levels and improvements in self‐decision‐making (Wang et al. 2024).

Contrastingly, a study providing lifestyle modification advice, follow‐up via telephone or home visits, and PFMT instruction using biofeedback found no significant differences in LUTS outcomes between the intervention and control groups (Robinson et al. 2008).

#### 3.5.3 Interventions Combining Lifestyle Modification and Symptom Management

Three studies reported interventions combining lifestyle modification with symptom management (Goode et al. 2011; Xu et al. 2025; Xin and Guo 2024). All three studies incorporated PFMT, with additional counselling (Xu et al. 2025) or biofeedback (Goode et al. 2011). Symptom management components included education on the causes of urinary incontinence, guidance focused on enhancing motivation and confidence to establish PFMT as a routine practice, and guidance on pad use. As a result, the intervention group demonstrated significantly greater improvements in urinary incontinence scores, anxiety and depression levels and self‐efficacy at 6 months postoperatively compared to the standard care group (Xin and Guo 2024). Furthermore, patients who received nursing interventions based on symptom management theory demonstrated significant reductions in urinary incontinence, pain, anxiety and depression compared with the control group, as well as significant improvements in QOL and reductions in postoperative complications (Xu et al. 2025).

In a study combining lifestyle modification with biofeedback‐assisted PFMT, significant improvements in urinary incontinence were observed from 2 months postoperatively onward compared with the control groups (Goode et al. 2011). However, no significant differences were observed between the lifestyle modification–only group and the group receiving combined lifestyle modification, biofeedback and PFMT (Goode et al. 2011).

#### 3.5.4 Interventions Combining Symptom Management and Follow‐Up Care

Online programmes or applications were used as support tools for symptom management. Specific components included weekly nurse‐led individualized assessment of participants' conditions, informational videos, guidance on individualized goal setting, monitoring of symptoms and tracking of PFMT practice (Heneka et al. 2024; Wennerberg et al. 2023).

In a single‐group study, significant improvements in irritative and obstructive urinary symptoms were observed at 3 months postoperatively compared with baseline (pre‐intervention); however, no significant differences were observed in urinary incontinence, nocturia or frequency (Heneka et al. 2024). Similarly, another single‐group study reported a significant improvement in urinary incontinence severity at 12 months postoperatively compared with 1, 3 and 6 months postoperatively; however, urinary incontinence severity did not return to the preoperative level (Wennerberg et al. 2023). Additionally, no significant differences were observed between the intervention and control groups (Wennerberg et al. 2023). In this study, application usage declined over time, suggesting challenges in maintaining long‐term symptom management (Wennerberg et al. 2023).

#### 3.5.5 Interventions Targeting Lifestyle Modification

Two studies reported self‐management interventions aimed at improving lifestyle habits, including PFMT (Gerlegiz et al. 2025; Yang et al. 2024). One study incorporated psychological counselling (Yang et al. 2024), which included establishing a trusting therapeutic relationship with patients, modifying maladaptive perceptions related to urinary incontinence, and providing examples of successful coping experiences to enhance patient confidence (Yang et al. 2024). No change was observed in the time required for improvement in urinary incontinence; however, improvements were reported in self‐care ability and knowledge acquisition at discharge, along with reductions in anxiety and depression scores.

A randomized controlled trial combining lifestyle modification and PFMT included training in the Knack manoeuvre, which involves a voluntary pre‐contraction of the pelvic floor muscles (Gerlegiz et al. 2025). Compared with the control group, the intervention group demonstrated significantly less urinary incontinence at 2 months postoperatively, along with improvements in the impact of incontinence, role limitations, physical limitations, restrictions in personal relationships and emotional problems (Gerlegiz et al. 2025).

## 4 Discussion

This scoping review provided an overview of self‐management interventions for LUTS following RP. Previous systematic reviews and meta‐analyses have examined the efficacy of conservative interventions and rehabilitation approaches for urinary incontinence following RP, as well as preoperative PFMT (Johnson et al. 2023; Zhou et al. 2023; Yu et al. 2024). These studies primarily aimed to evaluate the efficacy of individual interventions. In addition to this review‐based evidence, current international guidelines, including the AUA/GURS/SUFU and EAU guidelines, identify conservative approaches such as PFMT, patient education and counselling as key components of post‐prostate treatment symptom management. However, while both previous reviews and current guidelines clarify the role of conservative management, they provide limited detail on how these strategies are integrated into patients' daily lives and sustained as self‐management behaviours. This gap highlights the need to examine post‐RP LUTS interventions from a self‐management perspective. In contrast, this review mapped 17 studies of diverse interventions from a self‐management perspective—including physical and psychosocial aspects—and comprehensively organized their components, implementation methods and support strategies. This review complements existing guideline‐based and review‐based knowledge by clarifying the current status of self‐management interventions for LUTS following RP and identifying future research priorities.

The included studies were conducted across eight countries between 2008 and 2026, with 11 studies (64.7%) published within the past 5 years, indicating growing research interest in self‐management intervention. Nevertheless, substantial variability remains in intervention components and outcome measures, and few RCTs have used comparable outcome indicators to evaluate similar types of support, limiting direct cross‐study comparisons and precluding meta‐analytic synthesis. These findings highlight the need for greater standardization of intervention content and harmonization of outcome measures in future research.

The literature identified in this review did not consist of single, isolated techniques; interventions were typically composed of multiple support components, including lifestyle modification, follow‐up care and symptom management. Additionally, a range of complementary approaches, such as PFMT, physical training, biofeedback and psychological support, were often integrated. This multimodal structure suggests that these interventions are being flexibly and comprehensively tailored to individual patients to facilitate engagement in self‐management behaviours.

By categorizing the included interventions by intervention type and organizing outcomes according to these categories, this scoping review provides a comprehensive overview of the forms of support that may contribute to the promotion of self‐management behaviours among patients following RP. The review findings highlight self‐efficacy‐enhancing strategies, continuous professional support and behavioural reinforcement as intervention components warranting further investigation. Future well‐designed controlled studies should examine whether these components promote sustained self‐management behaviours and improve LUTS outcomes following RP.

Previous research includes a systematic review of preoperative PFMT, which concluded that effective implementation requires both patient engagement and continuous guidance from health care professionals (Zhou et al. 2023). Instruction in PFMT alone or lifestyle modification alone is often insufficient to sustain behaviour change or foster the self‐efficacy required for patients to perceive active control over their symptoms. However, a study investigating urinary incontinence in women reported that periodic monitoring by specialists, provision of continuous feedback and encouragement of voluntary practice could facilitate proficiency in PFMT (Sacomori et al. 2015). Collectively, these findings support the design of multifaceted self‐management interventions that integrate motivation, symptom monitoring, lifestyle adjustment and PFMT skill education within a framework that actively supports self‐efficacy.

Additionally, follow‐up care was commonly reported as a component of self‐management interventions that may support LUTS self‐management. This aligns with cancer survivorship literature emphasizing survivors' knowledge and skills for managing treatment‐related consequences and with oncology nursing literature highlighting educational conversations, symptom monitoring and person‐centred self‐management support (Girgis 2020; Bana et al. 2025). Systematic reviews of chronic pain self‐management similarly suggest that limited or absent post‐intervention support may hinder the continued use of self‐management strategies in daily life and that sustaining motivation can be a key challenge after completion of self‐management interventions (Devan et al. 2018; Hu et al. 2025). These findings may be relevant to self‐management intervention for LUTS in patients following RP, particularly digital programmes in which engagement may decline over time. In particular, some studies reported that specialized support extending beyond the formal intervention period may help patients adapt to long‐term changes in LUTS and maintain self‐management. Establishing a follow‐up system in advance, providing self‐management information from the pre‐discharge phase, making individualized adjustments to facilitate the integration of self‐management into daily activities, and promoting partnership‐based decision‐making may contribute to sustained behaviour change and improved self‐management practices.

Furthermore, this review suggests that PFMT combined with the Knack manoeuvre may provide greater improvement in subjective symptom severity compared to PFMT alone (Gerlegiz et al. 2025). Additionally, supportive accompaniment‐based interventions incorporating psychological counselling may represent a promising intervention component, based on evidence from a cluster randomized controlled trial in primary care demonstrating the effectiveness of continuous engagement combined with standardized educational materials (Drake et al. 2023). These approaches may support the self‐management of LUTS following RP. However, evidence regarding their long‐term outcomes remains limited, and further investigation is warranted to better characterize these outcomes.

LUTS following RP have been reported to improve over time as part of the postoperative course (Leyh‐Bannurah et al. 2021; Qvigstad et al. 2024). Consequently, rigorously distinguishing changes attributable to the natural postoperative trajectory from those resulting from the intervention itself is crucial to accurately interpreting the effects of these interventions. Therefore, reliance on simple pre–post comparisons is insufficient; instead, appropriately designed controlled studies employing stratified randomization are required to balance potential confounders such as time since surgery, LUTS severity, comorbidities and prior treatment history.

Moreover, considering the heterogeneity of interventions regarding intervention content and participant characteristics, accumulating evidence across multiple studies is necessary to better understand their reported outcomes. Such accumulation of evidence will enable more precise identification of the effectiveness of different forms of self‐management approaches for specific patients and conditions, thereby contributing to improved quality and consistency of support in nursing practice.

### 4.1 Strengths and Limitations

This review comprehensively maps and synthesizes evidence on support for self‐management of LUTS following RP, contributing to the existing evidence base by identifying gaps in the literature. Notably, studies focusing solely on conventional standard‐of‐care interventions for post‐RP patients, such as PFMT or biofeedback delivered in isolation, were intentionally excluded to specifically examine self‐management interventions beyond routine clinical practice. This review was conducted according to a protocol developed prior to the review process and originally written in Japanese. An English translation of the original protocol is provided in Appendices S1 and S2, together with a record of subsequent amendments and deviations documented to enhance transparency of the review process. A comprehensive and clearly defined search strategy, incorporating broad yet specific search terms, was applied alongside a systematic and transparent screening process to facilitate the identification of all relevant studies within urological care.

Another key strength of this scoping review is its comprehensive, cross‐cutting synthesis of self‐management approaches for LUTS in patients following RP. This review systematically collected and organized evidence spanning nurse‐led PFMT instruction, follow‐up care, psychosocial interventions and the use of supportive tools, integrating previously fragmented findings into a table‐based visual framework that is readily applicable to clinical practice.

Existing reviews have largely addressed isolated domains, such as PFMT alone, lifestyle guidance or device‐assisted interventions. Although short‐term effects have often been demonstrated, evidence regarding long‐term effectiveness and comparability across interventions remained limited (Benedetto et al. 2023; Johnson et al. 2023; Yu et al. 2024; Zhou et al. 2023). In contrast, the present review organizes intervention components, outcomes and adverse events using a consistent framework, thereby providing a structured overview to inform the consideration of interventions tailored to patients' needs and recovery stages.

This review included studies available up to July 2026. Although additional relevant studies may have been published after this period, it is unlikely that these publications would substantially alter the overall conclusions; nevertheless, some relevant intervention studies may not have been captured. The literature search was conducted using a comprehensive strategy across major databases commonly used in nursing and health sciences research, including PubMed, CINAHL, PsycINFO and Web of Science. Despite these efforts, not all existing databases were searched, and some relevant studies may not have been identified.

Additionally, this review included only studies published in English, which may have introduced the potential for language bias. Furthermore, eight of the included studies were conducted in China, indicating a geographical concentration of the evidence; consequently, the findings may not be generalizable to countries or regions with different cultural backgrounds or health care systems. Although the review was conducted in accordance with standard scoping review methodology, no assessment of the methodological quality or risk of bias of individual studies was included, representing an inherent limitation of this approach. Finally, because our synthesis prioritized LUTS‐specific outcomes, studies reporting only generic QOL without LUTS‐specific measures may have been underrepresented; moreover, the timing of intervention delivery was not restricted, which may increase heterogeneity and limit generalizability.

### 4.2 Implications for Nursing and/or Patient Care

This review suggests that nursing support for patients with LUTS following RP should emphasize helping patients maintain self‐management behaviours in daily life rather than focusing solely on symptom improvement. In particular, staged, achievable goals based on patients' daily needs and recovery process, developed collaboratively with patients, were identified as key intervention components. Furthermore, the reported self‐management interventions ranged from a single instructional session to ongoing support. Therefore, establishing a continuous support system that anticipates life after discharge, starting from the time of diagnosis, is warranted. Additionally, considering the course of LUTS varies from person to person, nurses must assess the patient's symptom management and respond in a manner that addresses the associated anxiety and decision‐making. Notably, since a loss of confidence due to symptom fluctuations or delayed improvement may lead to the discontinuation of self‐management, providing appropriate information while adjusting to the patient's level of understanding and offering support that enhances their sense of self‐efficacy are crucial aspects of this framework. These practices contribute to the establishment of patients' self‐management behaviours and the improvement of their QOL; therefore, the development of continuous and practical interventions tailored to individual needs is warranted in future nursing practice.

### 4.3 Synthesis Using the Patterns, Advances, Gaps, Evidence for Practice and Research Recommendations (PAGER) Framework

To provide an integrated synthesis of the above discussion, the findings of this review were organized using the PAGER framework (Bradbury‐Jones et al. 2022), which enabled a structured summary of key trends, developments, limitations and implications for practice and future research identified in the included studies (Table 3). Drawing together these domains, the PAGER framework highlights the multifaceted nature of self‐management interventions for LUTS following RP, while also identifying important gaps in current evidence and directions for future research. The PAGER framework therefore reinforcing the need for more standardized, theory‐based interventions and robust evaluation strategies, as well as highlighting the importance of follow‐up care in clinical practice.

**TABLE 3:** Practice and research implications.

	Pattern	Advances	Gaps	Evidence for practice	Research recommendations
1	Components of self‐management interventions for LUTS in post‐RP patients	Self‐management interventions for LUTS following RP have evolved into multi‐component interventions	The intervention design has not been standardized Among the components of the intervention, the details regarding lifestyle modifications are vague or insufficient	The studies included in this review frequently incorporated multiple self‐management components, such as lifestyle modification, symptom management and follow‐up care. This suggests that these components are commonly considered important for managing LUTS following RP	Standardization of self‐management intervention content Research to evaluate the effectiveness of individual components (component analysis) Design of theory‐based self‐management interventions
2	Evaluation framework for self‐management interventions for LUTS in patients following RP	A variety of assessment measures (such as lower urinary tract function, quality of life and psychological indicators) are used	Outcome measures are not standardized. Limited use of measures assessing patient self‐management	The included studies assessed a variety of outcomes beyond symptom improvement, including quality of life and psychological indicators, highlighting the multidimensional nature of outcome evaluation in self‐management interventions	RCTs using common outcome measures RCTs using assessment measures of patient self‐management
3	Findings on the effectiveness of self‐management interventions for LUTS in patients following RP	As the number of multi‐modal intervention studies increases, the scope of support is expanding beyond in‐person services to include remote support delivered via telephone, the internet and mobile applications	Insufficient comparison of the effects of different intervention combinations Variations in intervention intensity (frequency and duration) Uncertainty regarding the long‐term use of the applications	Follow‐up care was frequently incorporated into self‐management interventions to support sustained self‐management behaviours. Furthermore, interventions often combined multiple components, including strategies to enhance motivation, symptom monitoring, lifestyle modification and PFMT education, to promote self‐management and self‐efficacy	Comparative trials by intervention design Identification of optimal intervention combinations Research on optimizing intervention intensity and frequency Design and evaluation of intervention programmes tailored for clinical practice

Abbreviations: LUTS, lower urinary tract symptoms; RP, radical prostatectomy.

## 5 Conclusion

This scoping review mapped the range of self‐management intervention provided for LUTS in patients following RP and identified key gaps in the existing evidence base. Although the importance of postoperative self‐management support is increasingly recognized, substantial discrepancies remain between the content of support provided and the outcome measures used, with limited comparable intervention studies employing standardized LUTS‐specific outcome indicators.

This review identified strategies to enhance self‐efficacy and motivation, symptom monitoring, lifestyle adjustments and education on PFMT as key components of self‐management interventions. However, the evidence was inconsistent: while some studies reported improvements in primary LUTS outcomes, others found no significant between‐group differences or reported symptom worsening. Furthermore, rigorous comparative studies grounded in a theoretical framework and adequately controlling for confounding factors are necessary to clearly distinguish between symptom improvement resulting from the natural postoperative course and the effects of the intervention.

The accumulation of reproducible, theory‐based intervention studies aligned with the directions identified in this review will enable more precise identification of which intervention approaches may be most beneficial for different patients. Such advances are expected to contribute to the strengthening of LUTS self‐management support for patients following RP in nursing practice and service delivery.

## Author Contributions

**Koji Amano:** conceptualization, methodology, funding acquisition, visualization, writing – original draft, writing – review and editing, investigation, data curation, formal analysis. **Kumi Suzuki:** methodology, validation, data curation, visualization, writing – review and editing.

## Funding

This work was supported by Japan Society for the Promotion of Science, JP24K13820.

## Ethics Statement

The authors have nothing to report.

## Consent

The authors have nothing to report.

## Conflicts of Interest

The authors declare no conflicts of interest.

## Supporting information


**Appendix S1:** Search strategy.
**Appendix S2:** Data extraction instrument.

**Table S1:** PRISMA‐ScR checklist.

**Table S2:** Search terms in scoping reviews.

## Data Availability

All data supporting the findings of this scoping review are available within the article, its Supporting Information, and the published articles included in the review.
