# Supplementary material for: Self‐Management Interventions for Lower Urinary Tract Symptoms After Radical Prostatectomy: A Scoping Review of Intervention Components, Outcomes and Research Gaps
Source: Nurs Open. 2026 Nov 3;13(11):e70991. doi: 10.1002/nop2.70991 (PMC13731166; doi:10.1002/nop2.70991)
Supplement: Supplementary file 1 [file NOP2-13-e70991-s001.docx]

Supplementary Table 2: Search terms in scoping reviews

Date last searched: 6 July 2026

■	PubMed

|  | Query | Results |
| --- | --- | --- |
| #1 | (Prostatectomy) OR (Prostatectomies) OR (RALP) OR (RARP) OR (ORP) OR ("Prostatectomy"[Mesh]) | 55,988 |
| #2 | ("Prostate cancer") OR ("Prostatic Neoplasms") OR ("Prostate adenocarcinoma") OR ("Prostate tumor") OR ("Prostatic Neoplasms"[Mesh]) | 217,345 |
| #3 | (Surgery) OR ("Surgical Procedure") OR ("Surgical Procedures") OR ("Surgical Procedures, Operative"[Mesh]) | 6,423,860 |
| #4  (#2 & #3) | ((Surgery) OR ("Surgical Procedure") OR ("Surgical Procedures") OR ("Surgical Procedures, Operative"[Mesh])) AND (("Prostate cancer") OR ("Prostatic Neoplasms") OR ("Prostate adenocarcinoma") OR ("Prostate tumor") OR ("Prostatic Neoplasms"[Mesh])) | 66,684 |
| #5  (#4 or #1) | ((Surgery) OR ("Surgical Procedure") OR ("Surgical Procedures") OR ("Surgical Procedures, Operative"[Mesh])) AND (("Prostate cancer") OR ("Prostatic Neoplasms") OR ("Prostate adenocarcinoma") OR ("Prostate tumor") OR ("Prostatic Neoplasms"[Mesh])) OR ((Prostatectomy) OR (Prostatectomies) OR (RALP) OR (RARP) OR (ORP) OR ("Prostatectomy"[Mesh])) | 93,782 |
| #6 | (Self-management) OR ("Self management") OR (Selfmanagement) OR ("Self-Management"[Mesh]) OR (Self-care) OR ("Self-care") OR (Selfcare) OR ("Self-Care"[Mesh]) OR ("Symptom management") OR ("Symptom intervention") OR (Symptom-management) OR (Symptom-intervention) OR ("behavioral intervention") OR ("behavioral therapy") | 597,145 |
| #7 | (LUTS) OR ("Lower urinary tract symptoms") OR ("tract symptoms") OR ("Lower Urinary Tract Symptoms"[Mesh]) OR (LUTD) OR ("Lower urinary tract dysfunction") OR ("Lower urinary tract dysfunctions") OR ("Tract dysfunction") OR ("Urinary tract disorder") OR ("Storage symptoms") OR ("Storage symptom") OR ("Storage dysfunction") OR ("Overactive Bladder") OR ("Urinary Bladder, Overactive"[Mesh]) OR ("Urine incontinence") OR (Incontinence) OR ("Urinary incontinence") OR ("Urinary Incontinence"[Mesh]) OR ("Frequent micturition") OR ("Frequent urination") OR (Pollakisuria) OR ("Urinary frequency") OR ("Urinary urgency") OR (Nocturia) OR ("Nocturia"[Mesh]) | 101,808 |
| #8  (#5 & #6 & #7) | (((Surgery) OR ("Surgical Procedure") OR ("Surgical Procedures") OR ("Surgical Procedures, Operative"[Mesh])) AND (("Prostate cancer") OR ("Prostatic Neoplasms") OR ("Prostate adenocarcinoma") OR ("Prostate tumor") OR ("Prostatic Neoplasms"[Mesh])) OR ((Prostatectomy) OR (Prostatectomies) OR (RALP) OR (RARP) OR (ORP) OR ("Prostatectomy"[Mesh]))) AND ((Self-management) OR ("Self management") OR (Selfmanagement) OR ("Self-Management"[Mesh]) OR (Self-care) OR ("Self-care") OR (Selfcare) OR ("Self-Care"[Mesh]) OR ("Symptom management") OR ("Symptom intervention") OR (Symptom-management) OR (Symptom-intervention) OR ("behavioral intervention") OR ("behavioral therapy")) AND ((LUTS) OR ("Lower urinary tract symptoms") OR ("tract symptoms") OR ("Lower Urinary Tract Symptoms"[Mesh]) OR (LUTD) OR ("Lower urinary tract dysfunction") OR ("Lower urinary tract dysfunctions") OR ("Tract dysfunction") OR ("Urinary tract disorder") OR ("Storage symptoms") OR ("Storage symptom") OR ("Storage dysfunction") OR ("Overactive Bladder") OR ("Urinary Bladder, Overactive"[Mesh]) OR ("Urine incontinence") OR (Incontinence) OR ("Urinary incontinence") OR ("Urinary Incontinence"[Mesh]) OR ("Frequent micturition") OR ("Frequent urination") OR (Pollakisuria) OR ("Urinary frequency") OR ("Urinary urgency") OR (Nocturia) OR ("Nocturia"[Mesh])) | 351 |

■	CINAHL

|  | Query | Results |
| --- | --- | --- |
| #1 | MH "Prostatectomy+" OR prostatectomy OR "radical prostatectomy" OR prostatectomies OR RALP OR RARP OR ORP | 10,120 |
| #2 | MH "Prostatic Neoplasms+" OR "prostate cancer" OR "prostatic Neoplasms" OR "prostate adenocarcinoma" OR "prostate tumor" | 50,127 |
| #3 | MH "Surgery+" OR surgery OR "surgical procedure" OR "surgical procedures" OR operative | 725,591 |
| #4  (#2 & #3) | (MH "Prostatic Neoplasms+" OR "prostate cancer" OR "prostatic Neoplasms" OR "prostate adenocarcinoma" OR "prostate tumor") AND (MH "Surgery+" OR surgery OR "surgical procedure" OR "surgical procedures" OR operative) | 6,514 |
| #5  (#1 or #4) | (MH "Prostatectomy+" OR prostatectomy OR "radical prostatectomy" OR prostatectomies OR RALP OR RARP OR ORP) OR ((MH "Prostatic Neoplasms+" OR "prostate cancer" OR "prostatic Neoplasms" OR "prostate adenocarcinoma" OR "prostate tumor") AND (MH "Surgery+" OR surgery OR "surgical procedure" OR "surgical procedures" OR operative)) | 12,687 |
| #6 | "self management" OR self-management OR selfmanagement OR MH "Self Care+" OR "self care" OR self-care OR selfcare OR MH "Symptom Management+" OR "symptom management" OR "symptom intervention" OR symptom-management OR symptom-intervention OR "behavioral intervention" OR "behavioral therapy" | 120,393 |
| #7 | MH "Lower Urinary Tract Symptoms+" OR LUTS OR "lower urinary tract symptoms" OR "tract symptoms" OR LUTD OR "lower urinary tract dysfunction" OR "lower urinary tract dysfunctions" OR "tract dysfunction" OR MH "Urination Disorders+" OR "urinary tract disorder" OR "storage symptoms" OR "storage symptom" OR "storage dysfunction" OR MH "Overactive Bladder+" OR "overactive bladder" OR MH "Urinary Incontinence+" OR "urine incontinence" OR incontinence OR "urinary incontinence" OR "frequent micturition" OR "frequent urination" OR pollakisuria OR "urinary frequency" OR MH "Urinary Urgency" OR "urinary urgency" OR MH "Nocturia" OR nocturia | 46,176 |
| #8  (#5 & #6 & #7) | ((MH "Prostatectomy+" OR prostatectomy OR "radical prostatectomy" OR prostatectomies OR RALP OR RARP OR ORP) OR ((MH "Prostatic Neoplasms+" OR "prostate cancer" OR "prostatic Neoplasms" OR "prostate adenocarcinoma" OR "prostate tumor") AND (MH "Surgery+" OR surgery OR "surgical procedure" OR "surgical procedures" OR operative))) AND ("self management" OR self-management OR selfmanagement OR MH "Self Care+" OR "self care" OR self-care OR selfcare OR MH "Symptom Management+" OR "symptom management" OR "symptom intervention" OR symptom-management OR symptom-intervention OR "behavioral intervention" OR "behavioral therapy") AND (MH "Lower Urinary Tract Symptoms+" OR LUTS OR "lower urinary tract symptoms" OR "tract symptoms" OR LUTD OR "lower urinary tract dysfunction" OR "lower urinary tract dysfunctions" OR "tract dysfunction" OR MH "Urination Disorders+" OR "urinary tract disorder" OR "storage symptoms" OR "storage symptom" OR "storage dysfunction" OR MH "Overactive Bladder+" OR "overactive bladder" OR MH "Urinary Incontinence+" OR "urine incontinence" OR incontinence OR "urinary incontinence" OR "frequent micturition" OR "frequent urination" OR pollakisuria OR "urinary frequency" OR MH "Urinary Urgency" OR "urinary urgency" OR MH "Nocturia" OR nocturia) | 56 |

■	APA PsycInfo

|  | Query | Results |
| --- | --- | --- |
| #1 | DE "Prostatectomy" OR prostatectomy OR "radical prostatectomy" OR prostatectomies OR RALP OR RARP OR ORP | 737 |
| #2 | DE "Prostate Cancer" OR "prostate cancer" OR "prostatic Neoplasms" OR "prostate adenocarcinoma" OR "prostate tumor" | 3,967 |
| #3 | DE "Surgery" OR surgery OR DE "Surgical Procedure" OR "surgical procedure" OR "surgical procedures" OR operative | 77,023 |
| #4  (#2 & #3) | (DE "Prostate Cancer" OR "prostate cancer" OR "prostatic Neoplasms" OR "prostate adenocarcinoma" OR "prostate tumor") AND (DE "Surgery" OR surgery OR DE "Surgical Procedure" OR "surgical procedure" OR "surgical procedures" OR operative) | 570 |
| #5  (#1 or #4) | (DE "Prostatectomy" OR prostatectomy OR "radical prostatectomy" OR prostatectomies OR RALP OR RARP OR ORP) OR ((DE "Prostate Cancer" OR "prostate cancer" OR "prostatic Neoplasms" OR "prostate adenocarcinoma" OR "prostate tumor") AND (DE "Surgery" OR surgery OR DE "Surgical Procedure" OR "surgical procedure" OR "surgical procedures" OR operative)) | 1,030 |
| #6 | "self management" OR self-management OR selfmanagement OR DE "Self Care" OR "self care" OR self-care OR selfcare OR DE "Symptom Management" OR "symptom management" OR "symptom intervention" OR symptom-management OR symptom-intervention OR "behavioral intervention" OR "behavioral therapy" | 97,711 |
| #7 | DE "Lower Urinary Tract Symptoms" OR LUTS OR "lower urinary tract symptoms" OR "tract symptoms" OR LUTD OR "lower urinary tract dysfunction" OR "lower urinary tract dysfunctions" OR "tract dysfunction" OR DE "Urination Disorders" OR "urinary tract disorder" OR "storage symptoms" OR "storage symptom" OR "storage dysfunction" OR DE "Overactive Bladder" OR "overactive bladder" OR DE "Urinary Incontinence" OR "urine incontinence" OR incontinence OR "urinary incontinence" OR "frequent micturition" OR "frequent urination" OR pollakisuria OR "urinary frequency" OR "urinary urgency" OR DE "Nocturia" OR nocturia | 6,465 |
| #8  (#5 & #6 & #7) | ((DE "Prostatectomy" OR prostatectomy OR "radical prostatectomy" OR prostatectomies OR RALP OR RARP OR ORP) OR ((DE "Prostate Cancer" OR "prostate cancer" OR "prostatic Neoplasms" OR "prostate adenocarcinoma" OR "prostate tumor") AND (DE "Surgery" OR surgery OR DE "Surgical Procedure" OR "surgical procedure" OR "surgical procedures" OR operative))) AND ("self management" OR self-management OR selfmanagement OR DE "Self Care" OR "self care" OR self-care OR selfcare OR DE "Symptom Management" OR "symptom management" OR "symptom intervention" OR symptom-management OR symptom-intervention OR "behavioral intervention" OR "behavioral therapy") AND (DE "Lower Urinary Tract Symptoms" OR LUTS OR "lower urinary tract symptoms" OR "tract symptoms" OR LUTD OR "lower urinary tract dysfunction" OR "lower urinary tract dysfunctions" OR "tract dysfunction" OR DE "Urination Disorders" OR "urinary tract disorder" OR "storage symptoms" OR "storage symptom" OR "storage dysfunction" OR DE "Overactive Bladder" OR "overactive bladder" OR DE "Urinary Incontinence" OR "urine incontinence" OR incontinence OR "urinary incontinence" OR "frequent micturition" OR "frequent urination" OR pollakisuria OR "urinary frequency" OR "urinary urgency" OR DE "Nocturia" OR nocturia) | 6 |

■	Web of Science

|  | Query | Results |
| --- | --- | --- |
| #1 | ALL=(prostatectomy OR "radical prostatectomy" OR prostatectomies OR RALP OR RARP OR ORP) | 64,348 |
| #2 | ALL=("prostate cancer" OR "prostatic Neoplasms" OR "prostate adenocarcinoma" OR "prostate tumor") | 274,290 |
| #3 | ALL=(surgery OR "surgical procedure" OR "surgical procedures" OR operative) | 4,158,896 |
| #4  (#2 & #3) | ALL=(("prostate cancer" OR "prostatic Neoplasms" OR "prostate adenocarcinoma" OR "prostate tumor") AND (surgery OR "surgical procedure" OR "surgical procedures" OR operative)) | 42,987 |
| #5  (#1 or #4) | ALL=((prostatectomy OR "radical prostatectomy" OR prostatectomies OR RALP OR RARP OR ORP) OR (("prostate cancer" OR "prostatic Neoplasms" OR "prostate adenocarcinoma" OR "prostate tumor") AND (surgery OR "surgical procedure" OR "surgical procedures" OR operative))) | 94,674 |
| #6 | ALL=("self management" OR self-management OR selfmanagement OR "self care" OR self-care OR selfcare OR "symptom management" OR "symptom intervention" OR symptom-management OR symptom-intervention OR "behavioral intervention" OR "behavioral therapy") | 149,680 |
| #7 | ALL=(LUTS OR "lower urinary tract symptoms" OR "tract symptoms" OR LUTD OR "lower urinary tract dysfunction" OR "lower urinary tract dysfunctions" OR "tract dysfunction" OR "urinary tract disorder" OR "storage symptoms" OR "storage symptom" OR "storage dysfunction" OR "overactive bladder" OR "urine incontinence" OR incontinence OR "urinary incontinence" OR "frequent micturition" OR "frequent urination" OR pollakisuria OR "urinary frequency" OR "urinary urgency" OR nocturia) | 94,849 |
| #8  (#5 & #6 & #7) | ALL=(((prostatectomy OR "radical prostatectomy" OR prostatectomies OR RALP OR RARP OR ORP) OR (("prostate cancer" OR "prostatic Neoplasms" OR "prostate adenocarcinoma" OR "prostate tumor") AND (surgery OR "surgical procedure" OR "surgical procedures" OR operative))) AND ("self management" OR self-management OR selfmanagement OR "self care" OR self-care OR selfcare OR "symptom management" OR "symptom intervention" OR symptom-management OR symptom-intervention OR "behavioral intervention" OR "behavioral therapy") AND (LUTS OR "lower urinary tract symptoms" OR "tract symptoms" OR LUTD OR "lower urinary tract dysfunction" OR "lower urinary tract dysfunctions" OR "tract dysfunction" OR "urinary tract disorder" OR "storage symptoms" OR "storage symptom" OR "storage dysfunction" OR "overactive bladder" OR "urine incontinence" OR incontinence OR "urinary incontinence" OR "frequent micturition" OR "frequent urination" OR pollakisuria OR "urinary frequency" OR "urinary urgency" OR nocturia)) | 67 |
