# Supplementary material for: Self‐Management Interventions for Lower Urinary Tract Symptoms After Radical Prostatectomy: A Scoping Review of Intervention Components, Outcomes and Research Gaps
Source: Nurs Open. 2026 Nov 3;13(11):e70991. doi: 10.1002/nop2.70991 (PMC13731166; doi:10.1002/nop2.70991)
Supplement: Supplementary file 3 [file NOP2-13-e70991-s003.docx]

Scoping Review Protocol

Self-Management Interventions for Lower Urinary Tract Symptoms After Radical Prostatectomy: A Scoping Review of Intervention Components, Outcomes, and Research Gaps

Abstract

**Objective:** The objective of this scoping review is to map the existing literature on self-management interventions for lower urinary tract symptoms (LUTS) following radical prostatectomy (RP) and to describe their characteristics and reported outcomes.

**Introduction:** Lower urinary tract symptoms may occur following radical prostatectomy, and support programs incorporating self-management components have been developed for patients experiencing these symptoms. However, the characteristics of these interventions and their reported outcomes have not been comprehensively mapped.

**Inclusion criteria:** Eligible sources will include interventional and observational studies that examine or describe interventions incorporating self-management components for LUTS in adult men following RP.

**Methods:** This scoping review will follow the Arksey and O’Malley methodological framework. The databases searched included PubMed, CINAHL via EBSCOhost, APA PsycInfo via EBSCOhost, and Web of Science Core Collection via Clarivate. The initial searches were conducted in February 2025 and updated in July 2026. Data will be extracted, categorized, and synthesized based on intervention components.

**Keywords:** prostatectomy; lower urinary tract symptoms; self-management intervention; postoperative care

Introduction

Lower urinary tract symptoms (LUTS), including urinary incontinence, increased urinary frequency, and urinary urgency, are common following radical prostatectomy (RP) and may persist over the long term, adversely affecting patients’ quality of life and daily living (Hikita et al., 2022; Jones et al., 2023; Kohada et al., 2024; Sun & Gu, 2024). Although adherence to behaviors aimed at managing LUTS is important for successful treatment, patients may face difficulties in maintaining long-term self-management and obtaining sufficient support from health care professionals (Hodges et al., 2020; Sun & Gu, 2024). Studies of self-management interventions for LUTS following RP remain limited and heterogeneous, and the overall characteristics, components, and reported outcomes of these interventions have not been comprehensively mapped.

For the purposes of this review, a self-management intervention will be operationally defined as an intervention or support program designed to enhance patients’ active participation in managing LUTS following RP. Such interventions may include support for lifestyle modification, symptom monitoring and management, coping with LUTS and related challenges, and the initiation or maintenance of self-care behaviors. Conventional conservative management, such as PFMT, biofeedback, or supervised exercise delivered in isolation, will not be considered a self-management intervention unless it includes an additional component specifically intended to promote or sustain patients’ active self-management.

Review question

What is known about the components and reported outcomes of self-management interventions for lower urinary tract symptoms in patients following radical prostatectomy?

Inclusion criteria

Participants

This scoping review will include adult patients with prostate cancer who have undergone radical prostatectomy. Studies involving patients with benign prostatic hyperplasia, patients undergoing transurethral procedures, or patients who have not yet undergone radical prostatectomy, including those awaiting surgery, will be excluded.

Concept

The concept of interest will be self-management interventions or support programs targeting postoperative LUTS in patients following RP. For the purposes of this review, a self-management intervention will be operationally defined as an intervention or support program designed to enhance patients’ active participation in managing postoperative LUTS. Eligible interventions may include components supporting lifestyle modification, symptom monitoring and management, coping with LUTS and related challenges, and the initiation or maintenance of self-care behaviors.

Interventions consisting solely of conventional conservative management, such as PFMT, biofeedback, or supervised exercise delivered in isolation, will be excluded unless they include an additional component specifically intended to promote or sustain patients’ active self-management. The presence of professional supervision or follow-up alone will not be sufficient for an intervention to be classified as a self-management intervention. Studies focusing on additional therapies for postoperative LUTS, sexual dysfunction, or other post-treatment outcomes unrelated to the management of LUTS following RP will also be excluded. Interventions delivered exclusively before RP will be excluded.

Context

This scoping review will consider self-management interventions for postoperative lower urinary tract symptoms following radical prostatectomy across healthcare settings, including hospitals, outpatient clinics, community-based care, and home care. No restrictions will be placed on geographic location.

Types of sources

This scoping review will consider interventional studies, including randomized controlled trials and quasi-experimental designs, as well as observational studies examining self-management interventions for LUTS following RP. Only original research articles published in English will be considered. Methodological reports, narrative reviews, study protocols, conference abstracts, and commentaries will be excluded.

Methods

The proposed scoping review will be conducted using the methodological framework proposed by Arksey and O’Malley (Arksey & O’Malley, 2005). The results of the review will be reported in accordance with the Preferred Reporting Items for Systematic Reviews and Meta-Analyses Extension for Scoping Reviews (PRISMA-ScR) (Tricco et al., 2018).

Search strategy

The search strategy will aim to identify published original research studies examining self-management interventions for lower urinary tract symptoms among adult patients with prostate cancer who have undergone radical prostatectomy. The search strategy will be guided by the Population, Concept, Context framework. Search terms will include combinations of terms related to radical prostatectomy, prostate cancer, surgery, self-management, symptom management, self-care, and lower urinary tract symptoms.

The databases to be searched will include PubMed, CINAHL via EBSCOhost, APA PsycInfo via EBSCOhost, and Web of Science Core Collection via Clarivate. The initial searches were conducted in February 2025 and updated on 6 July 2026. The databases were searched from inception to the date of each search. Only original research articles published in English will be considered. Unpublished studies and gray literature will not be included. The complete search strategies for all databases will be provided in Appendix I.

Study/Source of evidence selection

Following the search, records retrieved from the four databases will be exported in CSV format and uploaded directly into Rayyan (Ouzzani et al., 2016). No other reference management software will be used. Duplicate candidates automatically identified by Rayyan will be manually reviewed by the first author (K.A.), and one record from each confirmed duplicate pair will be excluded.

Before screening, the first and second authors will discuss and agree on the eligibility and exclusion criteria. No pilot screening will be conducted. All identified records will be assessed against the eligibility criteria through title and abstract screening and full-text assessment. The literature screening will be conducted independently. The first author will screen all records, and the second author will verify the screening decisions. Any disagreements will be resolved through discussion and re-examination of the relevant articles until consensus is reached.

The exclusion criteria will be applied in three sequential stages. The first stage will assess eligibility based on language, publication type, and availability of the full text. The second stage will assess the study population and treatment received. The third stage will assess the outcomes, concept, and context of the study. When the full text of a potentially eligible article is not readily available, an attempt will be made to obtain it through interlibrary loan or other library services.

One primary reason for exclusion will be recorded for each excluded source. The results of the search and source selection process will be reported in the final scoping review and presented in a PRISMA flow diagram (Tricco et al., 2018).

Data extraction

Data will be extracted from the included studies by the first reviewer and verified by the second reviewer using a data charting form developed in Microsoft Excel (Appendix II). The extracted data will include: 1) publication details, including author(s), year of publication, and country; 2) study design, intervention components, intervention tools, outcome measures, and timing of assessment; 3) participant characteristics, including sample size, group allocation, age, prostate-specific antigen levels, clinical stage, nerve-sparing status, and other perioperative factors; and 4) intervention outcomes.

The components of self-management support and their associated outcomes will also be extracted to allow the findings to be organized according to each intervention component. Where information is unclear, the original article will be re-examined. Information that is not reported in the article will be recorded as “not reported,” and study authors will not be contacted for additional information.

A formal assessment of methodological quality or risk of bias will not be conducted because this is a scoping review.

Data analysis and presentation

Extracted data will be categorized according to the type of self-management intervention and the lower urinary tract symptoms addressed. The components of self-management support will be identified and categorized, and their relationships with the reported outcomes will be organized. The findings will be presented in tabular form. A narrative summary will accompany the tables and describe the characteristics, components, and reported outcomes of the interventions in relation to the review objective and question.

Acknowledgements

The authors have no acknowledgements to declare.

Funding

This work was supported by the Japan Society for the Promotion of Science (JSPS) KAKENHI (Grant Number JP24K13820).

Declarations

The authors have no declarations to report.

Author contributions

Koji Amano contributed to the conceptualization and design of the review and development of the search strategy, and will undertake the literature search, removal of duplicate records, study selection, data extraction, data analysis, and preparation of the review manuscript.

Kumi Suzuki contributed to the conceptualization and design of the review and will verify study selection and extracted data, contribute to the interpretation of the findings, and critically revise the review manuscript. Both authors reviewed and approved the final protocol.

Conflicts of interest

The authors declare that there are no conflicts of interest.

References

Arksey, H. & O’Malley, L. (2005). Scoping studies: towards a methodological framework. International Journal of Social Research Methodology, 8(1), 19–32. <https://doi.org/10.1080/1364557032000119616>

Hikita, K., Honda, M., Shimizu, R., Teraoka, S., Kawamoto, B., Yumioka, T., Tsounapi, P., Iwamoto, H., Morizane, S., & Takenaka, A. (2022). Longitudinal, 5-year long-term outcomes for urinary continence and quality of life after robot-assisted radical prostatectomy in Japanese patients. Lower urinary tract symptoms, 14(3), 178–185. <https://doi.org/10.1111/luts.12421>

Hodges, P. W., Stafford, R. E., Hall, L., Neumann, P., Morrison, S., Frawley, H., Doorbar-Baptist, S., Nahon, I., Crow, J., Thompson, J., & Cameron, A. P. (2020). Reconsideration of pelvic floor muscle training to prevent and treat incontinence after radical prostatectomy. Urologic oncology, 38(5), 354–371. <https://doi.org/10.1016/j.urolonc.2019.12.007>

Jones, C. P., Shaw, N. M., Mena, J., Breyer, B. N., Walter, L. C., Baussan, C., Quanstrom, K., Allen, I. E., Dohan, D., & Hampson, L. A. (2023). The relationship between frailty, incontinence severity, and treatment decisions for men with post-prostatectomy stress urinary incontinence: a mixed methods analysis. Translational andrology and urology, 12(5), 840–848. <https://doi.org/10.21037/tau-22-839>

Kohada, Y., Kitano, H., Tasaka, R., Miyamoto, S., Hatayama, T., Shikuma, H., Iwane, K., Yukihiro, K., Takemoto, K., Naito, M., Kobatake, K., Sekino, Y., Goto, K., Goriki, A., Hieda, K., & Hinata, N. (2024). Clinical characteristics and predictors of long-term postoperative urinary incontinence in patients treated with robot-assisted radical prostatectomy: A propensity-matched analysis. International journal of urology : official journal of the Japanese Urological Association, 31(10), 1145–1152. <https://doi.org/10.1111/iju.15533>

Ouzzani, M., Hammady, H., Fedorowicz, Z., & Elmagarmid, A. (2016). Rayyan: A web and mobile app for systematic reviews. Systematic Reviews, 5, 210. <https://doi.org/10.1186/s13643-016-0384-4>

Sun, N., & Gu, Y. (2024). Exploring the hidden struggles: a qualitative insight into urinary incontinence among prostate cancer survivors post-surgery. Patient preference and adherence, 18, 1047–1058. <https://doi.org/10.2147/PPA.S461027>

Tricco, A. C., Lillie, E., Zarin, W., O'Brien, K. K., Colquhoun, H., Levac, D., Moher, D., Peters, M. D. J., Horsley, T., Weeks, L., Hempel, S., Akl, E. A., Chang, C., McGowan, J., Stewart, L., Hartling, L., Aldcroft, A., Wilson, M. G., Garritty, C., Lewin, S., … Straus, S. E. (2018). PRISMA Extension for Scoping Reviews (PRISMA-ScR): Checklist and Explanation. Annals of internal medicine, 169(7), 467–473. https://doi.org/10.7326/M18-0850

Appendices

Appendix I: Search strategy

Initial search date: February 2025

Updated search date: 6 July 2026

■	PubMed

| **Search ID** | **Query** |
| --- | --- |
| #1 | (Prostatectomy) OR (Prostatectomies) OR (RALP) OR (RARP) OR (ORP) OR ("Prostatectomy"[Mesh]) |
| #2 | ("Prostate cancer") OR ("Prostatic Neoplasms") OR ("Prostate adenocarcinoma") OR ("Prostate tumor") OR ("Prostatic Neoplasms"[Mesh]) |
| #3 | (Surgery) OR ("Surgical Procedure") OR ("Surgical Procedures") OR ("Surgical Procedures, Operative"[Mesh]) |
| #4  (#2 & #3) | ((Surgery) OR ("Surgical Procedure") OR ("Surgical Procedures") OR ("Surgical Procedures, Operative"[Mesh])) AND (("Prostate cancer") OR ("Prostatic Neoplasms") OR ("Prostate adenocarcinoma") OR ("Prostate tumor") OR ("Prostatic Neoplasms"[Mesh])) |
| #5  (#4 or #1) | ((Surgery) OR ("Surgical Procedure") OR ("Surgical Procedures") OR ("Surgical Procedures, Operative"[Mesh])) AND (("Prostate cancer") OR ("Prostatic Neoplasms") OR ("Prostate adenocarcinoma") OR ("Prostate tumor") OR ("Prostatic Neoplasms"[Mesh])) OR ((Prostatectomy) OR (Prostatectomies) OR (RALP) OR (RARP) OR (ORP) OR ("Prostatectomy"[Mesh])) |
| #6 | (Self-management) OR ("Self management") OR (Selfmanagement) OR ("Self-Management"[Mesh]) OR (Self-care) OR ("Self-care") OR (Selfcare) OR ("Self-Care"[Mesh]) OR ("Symptom management") OR ("Symptom intervention") OR (Symptom-management) OR (Symptom-intervention) OR ("behavioral intervention") OR ("behavioral therapy") |
| #7 | (LUTS) OR ("Lower urinary tract symptoms") OR ("tract symptoms") OR ("Lower Urinary Tract Symptoms"[Mesh]) OR (LUTD) OR ("Lower urinary tract dysfunction") OR ("Lower urinary tract dysfunctions") OR ("Tract dysfunction") OR ("Urinary tract disorder") OR ("Storage symptoms") OR ("Storage symptom") OR ("Storage dysfunction") OR ("Overactive Bladder") OR ("Urinary Bladder, Overactive"[Mesh]) OR ("Urine incontinence") OR (Incontinence) OR ("Urinary incontinence") OR ("Urinary Incontinence"[Mesh]) OR ("Frequent micturition") OR ("Frequent urination") OR (Pollakisuria) OR ("Urinary frequency") OR ("Urinary urgency") OR (Nocturia) OR ("Nocturia"[Mesh]) |
| #8  (#5 & #6 & #7) | (((Surgery) OR ("Surgical Procedure") OR ("Surgical Procedures") OR ("Surgical Procedures, Operative"[Mesh])) AND (("Prostate cancer") OR ("Prostatic Neoplasms") OR ("Prostate adenocarcinoma") OR ("Prostate tumor") OR ("Prostatic Neoplasms"[Mesh])) OR ((Prostatectomy) OR (Prostatectomies) OR (RALP) OR (RARP) OR (ORP) OR ("Prostatectomy"[Mesh]))) AND ((Self-management) OR ("Self management") OR (Selfmanagement) OR ("Self-Management"[Mesh]) OR (Self-care) OR ("Self-care") OR (Selfcare) OR ("Self-Care"[Mesh]) OR ("Symptom management") OR ("Symptom intervention") OR (Symptom-management) OR (Symptom-intervention) OR ("behavioral intervention") OR ("behavioral therapy")) AND ((LUTS) OR ("Lower urinary tract symptoms") OR ("tract symptoms") OR ("Lower Urinary Tract Symptoms"[Mesh]) OR (LUTD) OR ("Lower urinary tract dysfunction") OR ("Lower urinary tract dysfunctions") OR ("Tract dysfunction") OR ("Urinary tract disorder") OR ("Storage symptoms") OR ("Storage symptom") OR ("Storage dysfunction") OR ("Overactive Bladder") OR ("Urinary Bladder, Overactive"[Mesh]) OR ("Urine incontinence") OR (Incontinence) OR ("Urinary incontinence") OR ("Urinary Incontinence"[Mesh]) OR ("Frequent micturition") OR ("Frequent urination") OR (Pollakisuria) OR ("Urinary frequency") OR ("Urinary urgency") OR (Nocturia) OR ("Nocturia"[Mesh])) |

■	CINAHL

| **Search ID** | **Query** |
| --- | --- |
| #1 | MH "Prostatectomy+" OR prostatectomy OR "radical prostatectomy" OR prostatectomies OR RALP OR RARP OR ORP |
| #2 | MH "Prostatic Neoplasms+" OR "prostate cancer" OR "prostatic Neoplasms" OR "prostate adenocarcinoma" OR "prostate tumor" |
| #3 | MH "Surgery+" OR surgery OR "surgical procedure" OR "surgical procedures" OR operative |
| #4  (#2 & #3) | (MH "Prostatic Neoplasms+" OR "prostate cancer" OR "prostatic Neoplasms" OR "prostate adenocarcinoma" OR "prostate tumor") AND (MH "Surgery+" OR surgery OR "surgical procedure" OR "surgical procedures" OR operative) |
| #5  (#1 or #4) | (MH "Prostatectomy+" OR prostatectomy OR "radical prostatectomy" OR prostatectomies OR RALP OR RARP OR ORP) OR ((MH "Prostatic Neoplasms+" OR "prostate cancer" OR "prostatic Neoplasms" OR "prostate adenocarcinoma" OR "prostate tumor") AND (MH "Surgery+" OR surgery OR "surgical procedure" OR "surgical procedures" OR operative)) |
| #6 | "self management" OR self-management OR selfmanagement OR MH "Self Care+" OR "self care" OR self-care OR selfcare OR MH "Symptom Management+" OR "symptom management" OR "symptom intervention" OR symptom-management OR symptom-intervention OR "behavioral intervention" OR "behavioral therapy" |
| #7 | MH "Lower Urinary Tract Symptoms+" OR LUTS OR "lower urinary tract symptoms" OR "tract symptoms" OR LUTD OR "lower urinary tract dysfunction" OR "lower urinary tract dysfunctions" OR "tract dysfunction" OR MH "Urination Disorders+" OR "urinary tract disorder" OR "storage symptoms" OR "storage symptom" OR "storage dysfunction" OR MH "Overactive Bladder+" OR "overactive bladder" OR MH "Urinary Incontinence+" OR "urine incontinence" OR incontinence OR "urinary incontinence" OR "frequent micturition" OR "frequent urination" OR pollakisuria OR "urinary frequency" OR MH "Urinary Urgency" OR "urinary urgency" OR MH "Nocturia" OR nocturia |
| #8  (#5 & #6 & #7) | ((MH "Prostatectomy+" OR prostatectomy OR "radical prostatectomy" OR prostatectomies OR RALP OR RARP OR ORP) OR ((MH "Prostatic Neoplasms+" OR "prostate cancer" OR "prostatic Neoplasms" OR "prostate adenocarcinoma" OR "prostate tumor") AND (MH "Surgery+" OR surgery OR "surgical procedure" OR "surgical procedures" OR operative))) AND ("self management" OR self-management OR selfmanagement OR MH "Self Care+" OR "self care" OR self-care OR selfcare OR MH "Symptom Management+" OR "symptom management" OR "symptom intervention" OR symptom-management OR symptom-intervention OR "behavioral intervention" OR "behavioral therapy") AND (MH "Lower Urinary Tract Symptoms+" OR LUTS OR "lower urinary tract symptoms" OR "tract symptoms" OR LUTD OR "lower urinary tract dysfunction" OR "lower urinary tract dysfunctions" OR "tract dysfunction" OR MH "Urination Disorders+" OR "urinary tract disorder" OR "storage symptoms" OR "storage symptom" OR "storage dysfunction" OR MH "Overactive Bladder+" OR "overactive bladder" OR MH "Urinary Incontinence+" OR "urine incontinence" OR incontinence OR "urinary incontinence" OR "frequent micturition" OR "frequent urination" OR pollakisuria OR "urinary frequency" OR MH "Urinary Urgency" OR "urinary urgency" OR MH "Nocturia" OR nocturia) |

■	APA PsycInfo

| **Search ID** | **Query** |
| --- | --- |
| #1 | DE "Prostatectomy" OR prostatectomy OR "radical prostatectomy" OR prostatectomies OR RALP OR RARP OR ORP |
| #2 | DE "Prostate Cancer" OR "prostate cancer" OR "prostatic Neoplasms" OR "prostate adenocarcinoma" OR "prostate tumor" |
| #3 | DE "Surgery" OR surgery OR DE "Surgical Procedure" OR "surgical procedure" OR "surgical procedures" OR operative |
| #4  (#2 & #3) | (DE "Prostate Cancer" OR "prostate cancer" OR "prostatic Neoplasms" OR "prostate adenocarcinoma" OR "prostate tumor") AND (DE "Surgery" OR surgery OR DE "Surgical Procedure" OR "surgical procedure" OR "surgical procedures" OR operative) |
| #5  (#1 or #4) | (DE "Prostatectomy" OR prostatectomy OR "radical prostatectomy" OR prostatectomies OR RALP OR RARP OR ORP) OR ((DE "Prostate Cancer" OR "prostate cancer" OR "prostatic Neoplasms" OR "prostate adenocarcinoma" OR "prostate tumor") AND (DE "Surgery" OR surgery OR DE "Surgical Procedure" OR "surgical procedure" OR "surgical procedures" OR operative)) |
| #6 | "self management" OR self-management OR selfmanagement OR DE "Self Care" OR "self care" OR self-care OR selfcare OR DE "Symptom Management" OR "symptom management" OR "symptom intervention" OR symptom-management OR symptom-intervention OR "behavioral intervention" OR "behavioral therapy" |
| #7 | DE "Lower Urinary Tract Symptoms" OR LUTS OR "lower urinary tract symptoms" OR "tract symptoms" OR LUTD OR "lower urinary tract dysfunction" OR "lower urinary tract dysfunctions" OR "tract dysfunction" OR DE "Urination Disorders" OR "urinary tract disorder" OR "storage symptoms" OR "storage symptom" OR "storage dysfunction" OR DE "Overactive Bladder" OR "overactive bladder" OR DE "Urinary Incontinence" OR "urine incontinence" OR incontinence OR "urinary incontinence" OR "frequent micturition" OR "frequent urination" OR pollakisuria OR "urinary frequency" OR "urinary urgency" OR DE "Nocturia" OR nocturia |
| #8  (#5 & #6 & #7) | ((DE "Prostatectomy" OR prostatectomy OR "radical prostatectomy" OR prostatectomies OR RALP OR RARP OR ORP) OR ((DE "Prostate Cancer" OR "prostate cancer" OR "prostatic Neoplasms" OR "prostate adenocarcinoma" OR "prostate tumor") AND (DE "Surgery" OR surgery OR DE "Surgical Procedure" OR "surgical procedure" OR "surgical procedures" OR operative))) AND ("self management" OR self-management OR selfmanagement OR DE "Self Care" OR "self care" OR self-care OR selfcare OR DE "Symptom Management" OR "symptom management" OR "symptom intervention" OR symptom-management OR symptom-intervention OR "behavioral intervention" OR "behavioral therapy") AND (DE "Lower Urinary Tract Symptoms" OR LUTS OR "lower urinary tract symptoms" OR "tract symptoms" OR LUTD OR "lower urinary tract dysfunction" OR "lower urinary tract dysfunctions" OR "tract dysfunction" OR DE "Urination Disorders" OR "urinary tract disorder" OR "storage symptoms" OR "storage symptom" OR "storage dysfunction" OR DE "Overactive Bladder" OR "overactive bladder" OR DE "Urinary Incontinence" OR "urine incontinence" OR incontinence OR "urinary incontinence" OR "frequent micturition" OR "frequent urination" OR pollakisuria OR "urinary frequency" OR "urinary urgency" OR DE "Nocturia" OR nocturia) |

■	Web of Science

| **Search ID** | **Query** |
| --- | --- |
| #1 | ALL=(prostatectomy OR "radical prostatectomy" OR prostatectomies OR RALP OR RARP OR ORP) |
| #2 | ALL=("prostate cancer" OR "prostatic Neoplasms" OR "prostate adenocarcinoma" OR "prostate tumor") |
| #3 | ALL=(surgery OR "surgical procedure" OR "surgical procedures" OR operative) |
| #4  (#2 & #3) | ALL=(("prostate cancer" OR "prostatic Neoplasms" OR "prostate adenocarcinoma" OR "prostate tumor") AND (surgery OR "surgical procedure" OR "surgical procedures" OR operative)) |
| #5  (#1 or #4) | ALL=((prostatectomy OR "radical prostatectomy" OR prostatectomies OR RALP OR RARP OR ORP) OR (("prostate cancer" OR "prostatic Neoplasms" OR "prostate adenocarcinoma" OR "prostate tumor") AND (surgery OR "surgical procedure" OR "surgical procedures" OR operative))) |
| #6 | ALL=("self management" OR self-management OR selfmanagement OR "self care" OR self-care OR selfcare OR "symptom management" OR "symptom intervention" OR symptom-management OR symptom-intervention OR "behavioral intervention" OR "behavioral therapy") |
| #7 | ALL=(LUTS OR "lower urinary tract symptoms" OR "tract symptoms" OR LUTD OR "lower urinary tract dysfunction" OR "lower urinary tract dysfunctions" OR "tract dysfunction" OR "urinary tract disorder" OR "storage symptoms" OR "storage symptom" OR "storage dysfunction" OR "overactive bladder" OR "urine incontinence" OR incontinence OR "urinary incontinence" OR "frequent micturition" OR "frequent urination" OR pollakisuria OR "urinary frequency" OR "urinary urgency" OR nocturia) |
| #8  (#5 & #6 & #7) | ALL=(((prostatectomy OR "radical prostatectomy" OR prostatectomies OR RALP OR RARP OR ORP) OR (("prostate cancer" OR "prostatic Neoplasms" OR "prostate adenocarcinoma" OR "prostate tumor") AND (surgery OR "surgical procedure" OR "surgical procedures" OR operative))) AND ("self management" OR self-management OR selfmanagement OR "self care" OR self-care OR selfcare OR "symptom management" OR "symptom intervention" OR symptom-management OR symptom-intervention OR "behavioral intervention" OR "behavioral therapy") AND (LUTS OR "lower urinary tract symptoms" OR "tract symptoms" OR LUTD OR "lower urinary tract dysfunction" OR "lower urinary tract dysfunctions" OR "tract dysfunction" OR "urinary tract disorder" OR "storage symptoms" OR "storage symptom" OR "storage dysfunction" OR "overactive bladder" OR "urine incontinence" OR incontinence OR "urinary incontinence" OR "frequent micturition" OR "frequent urination" OR pollakisuria OR "urinary frequency" OR "urinary urgency" OR nocturia)) |

### Appendix II: Data extraction instrument

| **Category** | **Data item** |
| --- | --- |
| Publication details | Author(s) |
| Publication details | Year of publication |
| Publication details | Country |
| Study characteristics | Study design |
| Intervention characteristics | Intervention components |
| Intervention characteristics | Intervention tools |
| Outcome assessment | Outcome measures |
| Outcome assessment | Timing of assessment |
| Participant characteristics | Sample size |
| Participant characteristics | Group allocation |
| Participant characteristics | Age |
| Participant characteristics | Prostate-specific antigen levels |
| Participant characteristics | Clinical stage |
| Participant characteristics | Nerve-sparing status |
| Results | Intervention outcomes |

Amendment and Deviation Log

The following log summarizes substantive amendments, clarifications, and deviations from the original protocol, including revisions made following peer review. Minor editorial revisions that did not affect the conduct or interpretation of the review are not listed.

| **No.** | **Protocol section** | **Original protocol** | **Revised protocol** | **Classification** | **Rationale** | **Timing** |
| --- | --- | --- | --- | --- | --- | --- |
| 1 | Title; Objective; Review question; Data analysis and presentation | The title, objective, review question, and data presentation plan referred to the effectiveness of self-management interventions. | References to effectiveness were replaced with reported outcomes, and the review focus was reframed as the descriptive mapping of intervention components and reported outcomes. | Amendment and methodological clarification | To align the terminology with the descriptive purpose of a scoping review. | Following peer review |
| 2 | Introduction; Inclusion criteria: Concept | No explicit operational definition of self-management intervention was provided. | An operational definition was added, and the distinction from conventional conservative management was clarified. | Clarification | To ensure consistent application of the Concept criterion. | Following peer review |
| 3 | Abstract: Inclusion criteria; Types of sources | The Abstract referred only to randomized and quasi-experimental designs, whereas the Methods also included observational studies. | The Abstract was revised to specify that both interventional and observational studies were eligible. | Correction and clarification | To resolve an internal inconsistency. | Following peer review |
| 4 | Inclusion criteria: Participants and Concept | The protocol included patients who had undergone RP and excluded patients who had not yet undergone RP, but the eligibility of studies involving preoperative interventions was not explicitly described. | The criteria were clarified to specify that studies focusing on patients who had not yet undergone RP, including those awaiting surgery, and interventions delivered exclusively before RP were not eligible. | Clarification and correction in study selection | To ensure transparent and consistent application of the eligibility criteria to studies involving preoperative patients or interventions. | Following peer review |
| 5 | Search strategy; Appendix I | The original search strategy included terms related to RP, prostate cancer, surgery, self-management, self-care, symptom management, and LUTS, with an initial search planned through February 2025. | The database-specific search strategies were refined, including the addition of terms related to behavioral interventions and behavioral therapy. The searches were updated in July 2026. | Amendment to the search strategy and updated search | To broaden the self-management concept block, improve the database-specific search syntax, and identify potentially eligible studies described using behavioral-intervention terminology. | Before the updated search in July 2026 |
